# Hydride Formation and Decomposition on Cu(111) in HClO_4_

**DOI:** 10.1021/jacs.4c12782

**Published:** 2025-01-27

**Authors:** David Raciti, Thomas P. Moffat

**Affiliations:** Materials Science and Engineering Division, National Institute of Standards and Technology, 100 Bureau Drive, Gaithersburg, Maryland 20899, United States

## Abstract

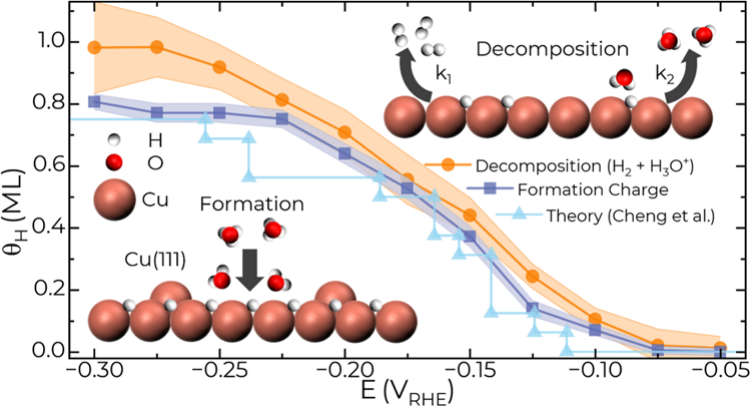

Cu electrodeposition and the electrocatalysis of hydrogenation reactions thereupon involve significant interactions with adsorbed hydrogen. Electrochemical mass spectrometry (EC-MS) is used to explore the formation and decomposition of surface hydride on Cu(111) in 0.1 mol L^–1^ HClO_4_. Hydride formation is associated with two reduction waves that reflect the potential-dependent H_ads_ coverage and its reconstruction. Voltammetric cycling reveals an additional oxidative and reductive feature at ≈ −0.05 V versus the reversible hydrogen electrode (RHE) that reflects the state of the 2D surface hydride. Extending the voltammetric window to more negative potentials results in an increase in H_ads_ coverage and surface reconstruction that subsequently leads to accelerated hydride decomposition at positive potentials. Voltammetric and chronoamperometric analysis of hydride formation indicates a H_ads_ coverage of ≈0.75 monolayers (ML) between −0.225 V vs RHE and −0.275 V vs RHE with further increases in H_ads_ observed with the onset and acceleration of the HER at more negative potentials. Returning to more positive potentials, hydride decomposition begins above −0.05 V vs RHE. Recombination of H_ads_ to form H_2_ accounts for desorption of ≈0.5 ML of H_ads_ while its oxidation to H_3_O^+^ consumes between ≈0.15 and ≈0.4 ML of H_ads_, depending on the specific electrochemical conditions. The potential-dependent H_ads_ coverage and surface reconstruction are congruent with trends identified in recent computational and electrochemical scanning tunneling microscopy studies. In contrast to perchloric acid, the presence of strongly adsorbing anions, such as sulfate or halides, favors hydride decomposition via the recombination pathway.

## Introduction

The technological impact of Cu spans a wide range from its ubiquitous presence in microelectronic interconnects to its use as a catalyst in emergent electrosynthetic reactions relevant to the electrification of the chemical industry.^1−5^ In these applications, the interaction of Cu with hydrogen exerts important effects that need to be understood. In electrolytic and electroless Cu deposition, hydrogen can be incorporated within growing films, thereby impacting their physical properties and even stimulating room temperature recrystallization.^6−11^ Under more aggressive conditions, cogeneration of hydrogen gas during electrodeposition can be utilized to template the growth of 3-D Cu foams for use in thermal management, bonding, and electrocatalysis.^12^ Excursions to such negative potentials can also lead to cathodic corrosion that is likely related to hydride formation.^13^

Of particular interest is the finding that Cu is the only elemental electrocatalyst capable of efficiently reducing CO_2_ to hydrocarbons and oxygenates. In a related fashion, Cu–H functionalized molecules have a long history in organic synthesis as potent reaction centers for CO_2_ reduction.^14−16^ For such multistep hydrogenation reactions, competitive and coadsorption between CO, H, and other reaction intermediates play a central role and likewise are known to induce and guide potential-dependent mesoscale rearrangements of Cu surfaces.^13−15,17−20^ The interaction of CO with Cu surfaces has received substantial attention, while the role of adsorbed hydrogen and its interactions with CO in various hydrogenation reactions remains understudied and unresolved.^21^ It is also noteworthy that some years ago UHV studies of hydrogenation reactions on hydrogen-dosed surfaces demonstrated that subsurface hydrogen species can serve as active intermediates although the possibility of such pathways has received limited attention in electrochemical systems.^22−24^ Studies of different synthetic pathways for producing bulk crystalline CuH yield different surface properties that speak to the complexity of the system and the need for further study.^25^ UHV studies of hydrogen adsorption on low-index Cu surfaces reveal the formation of two-dimensional surface hydride superlattices and also provide the earliest measurement of competitive adsorption dynamics between H and CO albeit at low temperatures.^26−32^ More recently, operando methods like electrochemical scanning tunneling microscopy (ECSTM), shell-isolated nanoparticle enhanced Raman spectroscopy (SHINERS), and electrochemical mass spectrometry (EC-MS) have been utilized to study H adsorption on copper surfaces in electrolytic environments.^33−37^ A complex, but stochiometric, (4 × 4) hydride overlayer structure was imaged on Cu(111) in sulfuric acid.^33,34^ Meanwhile EC-MS measurements indicate a potential regime where a saturated hydride phase is formed with a coverage of (0.67 ± 0.15) monolayers (ML) in 0.1 mol L^–1^ H_2_SO_4_.^36^ Asymmetric voltammetry arises from the slow formation of the hydride phase by proton reduction with development of a ≈1040 cm^–1^ vibrational band during the negative-going scan. Its subsequent desorption by hydrogen recombination occurs on the positive-going scan that is stimulated by anion adsorption (sulfate band at 1200 cm^–1^).^35^ Recent atomistic simulations indicate that hydrogen-induced reconstruction of Cu(111) creates Cu adatoms that facilitate both the hydrogen evolution reaction (HER) and CO_2_/CO reduction in acidic environments subject to competitive interactions in the operational environment.^33,38,39^

In the present study, the formation of surface hydride on Cu(111) in 0.1 mol L^–1^ HClO_4_ is examined with proton reduction partitioned between hydride formation and HER. Voltammetric EC-MS measurements reveal at least two electrochemical peaks related to surface hydride formation on Cu(111), that involve populating different surface sites and/or phase transitions and reconstructions, while subsequent hydride decomposition at more positive potentials is associated with a single voltammetric wave. In contrast to studies with sulfate (SO_4_^2–^) or chloride (Cl^–^), perchlorate ions (ClO_4_^–^) are not expected to specifically adsorb on copper surfaces, yet measurable oxidative current is still observed at potentials that overlap with hydride decomposition at higher potentials.^35,40^ Chronoamperometric EC-MS measurements were employed to quantify the H_ads_ coverage with particular attention given to partitioning the hydride decomposition between H_ads_ recombination versus electrochemical oxidation back to hydronium.

## Experimental Section

### Preparation of the Cu(111) Electrode

Before EC-MS measurements were conducted, the 5 mm diameter Cu(111) crystal was mechanically polished using an alumina slurry to achieve a 0.05 μm finish. This was followed by rinsing and sonicating the specimen in water multiple times to eliminate any remaining alumina particles. Subsequently, the Cu(111) disk was electropolished for 5 min at +1.6 V vs a Pt counter electrode, in 85% H_3_PO_4_. The Cu(111) surface was positioned facing upward and opposite of the gas-generating Pt gauze that was ≈6 cm away. Following ≈10 min of electropolishing, the disk was rinsed and then shielded with H_2_O until being dried with flowing Ar immediately before being mounted in the mass spectrometer.

### Electrochemical Mass Spectrometry Measurements

EC-MS was performed using a thin layer cell configuration (Spectro Inlets*).^41,42^ Experiments were performed in freshly prepared Ar purged 0.1 mol L^–1^ HClO_4_ electrolyte (70% HClO_4_, 99.999% trace metal basis from Sigma-Aldrich). Glass side arms on opposite sides of the working electrode contained an Ir wire counter electrode and a capillary consisting of a Pt wire submerged in H_2_ saturated electrolyte to form a trapped H_2_ bubble quasi-reversible hydrogen reference electrode (RHE), respectively. The Ir counter electrode was placed >3 cm away from the entrance to the working electrode compartment to minimize any contribution from the counter-reactions. To mitigate oxidation and dissolution of the Cu(111) working electrode that will occur under open circuit conditions, potentiostatic control at 0 V vs RHE (to be assumed throughout) was imposed immediately following wetting of the three electrodes by the electrolyte. For subsequent rest or idle periods between electrochemical measurements, the working electrode was typically poised at 0.175 V. A Biologic SP-200 potentiostat was used for all electrochemical measurements. The EC-MS and electrochemical data were collected on the same computer enabling effective time synchronization between the two measurements. For cyclic voltammetry measurements, the baseline of the 2 amu EC-MS signal was subtracted using a linear profile with the slope determined by averaging the signal over 2 s before and after completion of the voltammetric scan.

For negative step potential pulse measurements, the 2 amu baseline was zeroed by averaging the signal for ≈2 s prior to initiating each potential step (*E*_Pulse_) (Figure S1). The approach is congruent with the absence of steady-state HER occurring at the rest potential of 0.175 V (*E*_Rest_). For positive step potential pulse measurements, determining the background for the 2 amu H_2_ measurement is more difficult. Fortunately, extended observation of the MS baseline drift indicates that, absent an obvious input source, the baseline for 32 amu (O_2_) and 2 amu (H_2_) change at the same rate. Accordingly, the 32 amu signal is used to provide an estimate of the drift in the H_2_ background levels. The utility of this alignment is evident in Figure S2 where the drift in the 32 amu signal was used to estimate the baseline signal for 2 amu (red line). It is noteworthy that when *E*_Rest_ > 0 V, where no HER occurs, the 2 amu signal drops to the baseline, overlapping the 32 amu baseline signal, supporting this strategy.

The 2 amu signal was converted to H_2_ flux following the strategy discussed previously.^36^ In short, calibration curves were generated under steady-state HER conditions that use the current, converted to H_2_ flux, to calibrate the 2 amu signal using either Cu or Pt electrodes. The electrolyte thickness separating the working electrode from the membrane entrance to the quadrupole mass spectrometer was determined using impulse measurements discussed previously and was typically found to be ≈120 μm.^36,43^

Impedance measurements of Cu(111) mounted in the EC-MS were conducted in 0.1 mol L^–1^ HClO_4_ using a 10 mV perturbation with a range of 100 mHz to 100 kHz using 10 points per decade. Measurements were performed as the potential was advanced from 0.2 to −0.25 V in 50 mV increments. The Nyquist plots (Figure S3) were fit to a simplified Randle circuit, and the results are presented in Table S1.

## Results and Discussion

### Cyclic Voltammetry on Cu(111) in 0.1 mol L^–1^ HClO_4_

**Figure 1:**
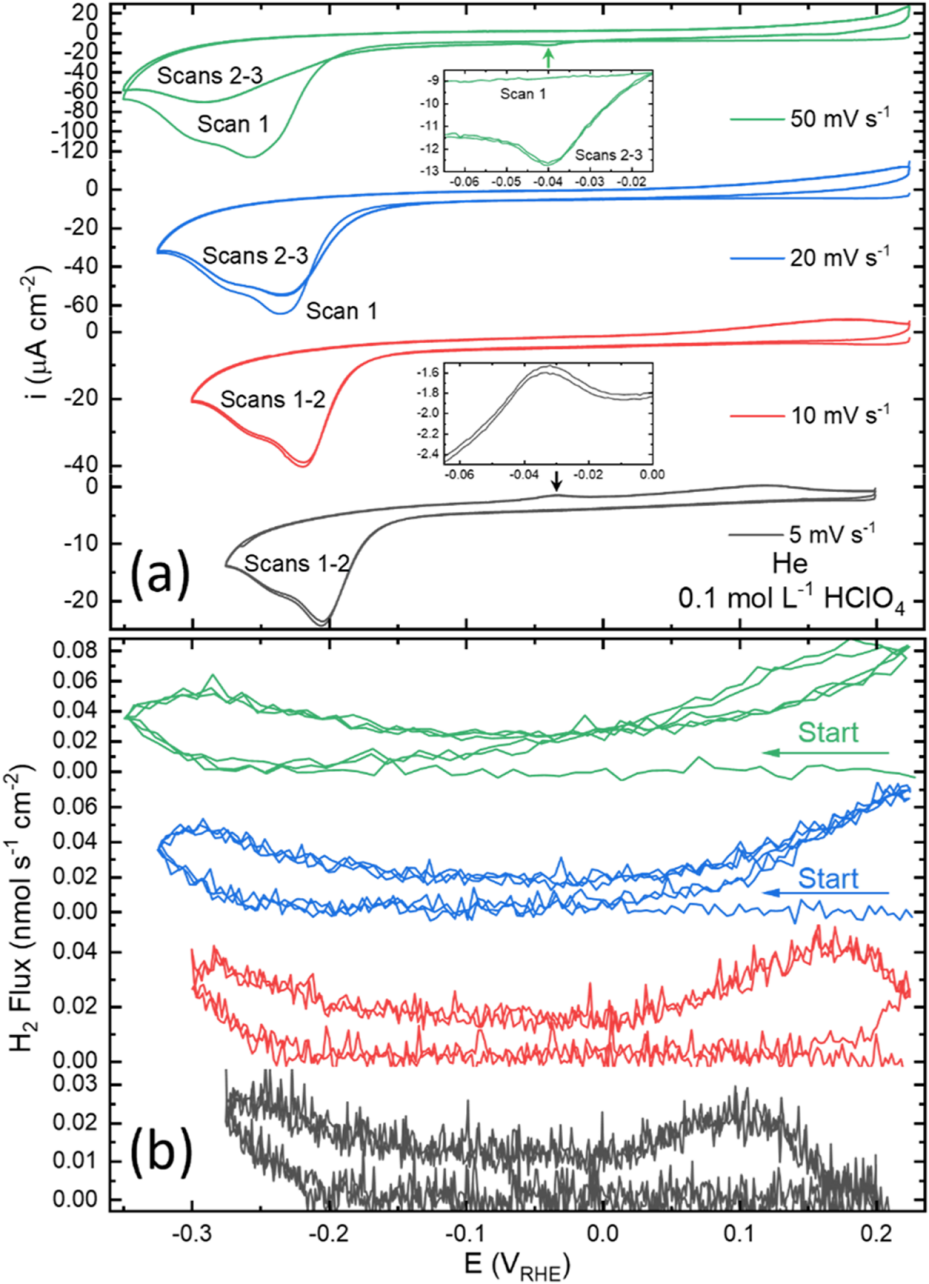
(a) Voltammetry and (b) 2 amu H_2_ flux measured at different scan rates in the thin layer EC-MS cell reveal hydride formation and decomposition for Cu(111) in 0.1 mol L^–1^ HClO_4_.

Recent EC-MS studies have revealed the formation of surface hydride on Cu(111) in various acid electrolytes.^35^ For 0.1 mol L^–1^ HClO_4_, hydride formation is associated with two overlapping voltammetric reduction peaks, e.g., at −0.22 and −0.26 V for 10 mV s^–1^, in Figure 1a while only a single peak is observed in electrolytes containing SO_4_^2–^, Cl^–^ and PO_4_^3–^. The origin of the two voltammetric peaks in perchloric acid is unresolved although recent theoretical work on H adsorption on Cu(111) indicates a series of phase transitions with increasing H coverage.^33,38^ For a pH of 1, an equilibrium H coverage of 0.56 ML is predicted between −0.200 and −0.241 V followed by an increase in coverage to 0.75 ML between −0.250 and −0.426 V coincident with the development of (4 × 4) superlattice seen in EC-STM experiments.^34,40^ At more negative potentials, surface reconstruction occurs with the formation of an ordered (4 × 4) array of Cu adatoms stabilized by the higher H coverages >0.94 ML. It should be noted that the computational uncertainties with the DFT exchange correlation functional, and the solvation and electrolyte models are such that the stated potentials could be off by up to ±0.3 V.^33^

**Figure 2:**
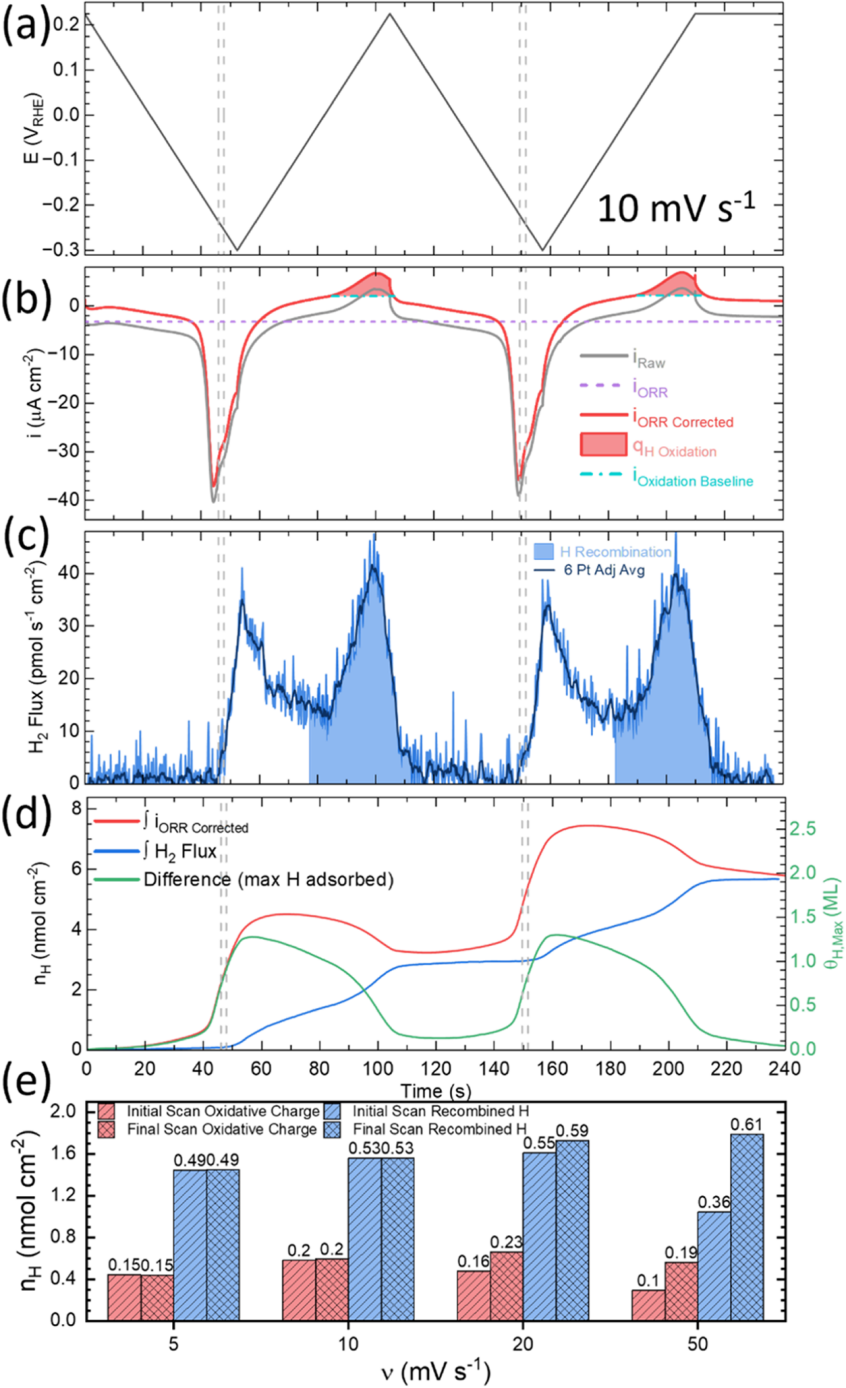
Traces of (a) potential, (b) current, and (c) H_2_ flux vs time from 10 mV s^–1^ voltammetry shown in Figure 1. (d) Integration of (b) current and (c) H_2_ flux as well as their difference in the form of atomic hydrogen (mol cm^–2^). (e) Decomposition of the hydride is quantified by the charge associated with oxidation of H_ads_ to H_3_O^+^ (shaded red in (b)) and the amount of H_2_ produced by recombination (shaded blue in (c)). The analyses for the initial and final cycle at different scan rates in Figure 1 are summarized (e) with the fractional H_ads_ surface coverage indicated on the top of the respective bars. The group of two dashed lines indicates the potential region for onset of HER.

Variation of the scan rate from 5 to 50 mV s^–1^ induces a ≈ −50 mV shift in the reduction wave peak potentials indicative of a kinetic constraint on surface hydride formation (Figure 1). This shift is not due to ohmic losses, as EIS measurements reveal the solution resistance to be 20.6 ± 0.6 Ohms (see Figure S3 and Table S1), which would result in <1 mV shift in the peak potential for the peak current measured in the 50 mV/s scan. Continued voltammetric scanning at rates ≥20 mV s^–1^ reveals that hydride decomposition is not completed during the first cycle, and as a result, the peak proton reduction current to reform the hydride during subsequent cycles is noticeably diminished (Figure 1a). Additionally, a small reduction peak develops near −0.05 V, which is associated with the incomplete removal of the hydride phase during the first voltammetric cycle. The magnitude of the peak at −0.05 V increases with scan rate, when cycling without interruption, as shown in Figure S4. During the return scan to positive potentials, the EC-MS reveals evolution of H_2_ at potentials above 0.0 V due to the decomposition of the hydride phase by H_ads_ recombination (Figure 1b) with the onset and rate of the decomposition being sensitive to the presence of anions and more specifically their strength of adsorption.^35^ Strongly adsorbing Cl^–^ shifts hydride decomposition to potentials negative of 0.0 V, Figure S5, while the much weaker interaction with ClO_4_^–^ is such that the H_ads_ combination process is still underway at the upper vertex at 0.2 V. The final increments of H_ads_ do not leave the surface until the second negative-going scan as evidenced by the measured H_2_ flux (Figure 1b), better visualized when plotted vs time (Figure 2). For faster scan rates, even less decomposition of hydride occurs by the end of the first cycle (Figures 1b, S6, and S7). Detection of H_2_ from recombination is apparent at potentials > −0.05 V (Figure 1b) with the final increments that are detected on the negative scan reflecting the slow kinetics of decomposition and diffusional lag in H_2_ collection.

An alternative pathway to hydride decomposition by recombination is oxidation to H_3_O^+^. As an upper bound the measured anodic charge, denoted q_H Oxidation_, of (48 ± 14) μC cm^–2^ for this process corresponds on average to removal Δθ_H_ of (0.17 ± 0.05) ML of H_ads_ (Figure 2b,e) following eq 1,
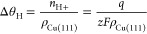
1where q is the charge density (C/cm^2^), *z* is the number of electrons transferred (mol e^–^/mol H), *F* is Faraday’s constant (C/mol e^–^), *n*_H+_ is the moles of H^+^ produced (mol H/cm^2^), and ρ_Cu(111*)*_ is the atomic surface density of Cu(111) which is 2.935 nmol/cm^2^.

The possibility that H_ads_ displacement by recombination to H_2_ is driven by the adsorption of trace anion contaminants was evaluated. The most likely contaminants are Cl^–^ and PO_4_^3–^ with the former being an impurity in the as-received perchloric acid and the latter being carried over from electropolishing. Voltammetry, at 5 mV s^–1^ following titration of 0.1 mol L^–1^ HClO_4_ with 100 μmol L^–1^ HCl, reveals that Cl^–^ adsorption drives decomposition of the hydride phase by H_ads_ recombination, which peaks at −0.08 V before reaching completion below 0 V (Figure S5a). Halide addition also shifts hydride formation to more negative potentials, with the peak current only being reached near −0.3 V as the adsorbed Cl^–^ must desorbed before the hydride can form. For a more dilute Cl^–^ concentration of 10 μmol L^–1^ (Figure S5b), halide adsorption and hydride decomposition shift to more positive potentials. Both the current and EC-MS peaks split into two broad but distinguishable events. The first is centered near −50 mV with the second near +75 mV with the peak separation reflecting the mixed control kinetics associated with the transport-limited halide flux relative to the scan rate. In contrast, Cl^–^ desorption and hydride formation on the negative-going scan appear as a single wave, indicating a saturated halide surface coverage is already present at this point in the experiment. The hydride formation peak shifts slightly positive to −0.275 V consistent with thermodynamic expectations for Cl^–^ desorption at the more dilute concentration. Finally, for the 1 μmol L^–1^ Cl^–^ titration both the voltammetry and EC-MS H_2_ flux profile (Figure S5c) are similar to that in neat 0.1 mol L^–1^ HClO_4_ although some differences remain. During the positive sweep the onset of Cl^–^ adsorption between −50 and −25 mV is followed by mass transport-limited accumulation which displaces the hydride at a fixed rate of ≈ 0.15 nmol s^–1^ cm^–2^ that is sustained to the upper potential limit. In contrast, in Cl^–^ free solution (Figure 1a), the primary anodic wave and hydride decomposition peak at 5 mV s^–1^ occur at a much more positive potential near +0.1 V. A small oxidative adsorption wave is evident near ≈ −0.04 V that reflects some change in the hydride phase prior to the onset of its decomposition. Interestingly, a similar peak develops in Cl^–^ containing media that was ascribed to a phase transition in the Cl^–^ adlayer however in that experiment the feature only appears after multiple voltammetric cycles.^44,45^ Further work will be required to understand the nature of this small feature.

Integration of the voltammetric current and H_2_ flux, following baseline subtraction and conversion of the 2 amu signal (see the Experimental Section), provides an avenue to track H coverage with time and assess the total collection ability of the thin layer EC-MS cell as summarized in Figure 2. Care must be taken in evaluating the baseline and contributions from possible background reactions, such as residual O_2_ reduction. For fast scan rates and low Faradaic currents, the electrode charging capacitance is significant but largely nulled by integration over each complete voltammetric cycle. At slower scan rates, the parasitic current due to transport-limited reduction of residual O_2_ is evident in the background offset of the voltammetric current. For scan rates <10 mV/s this is compensated by subtracting a fixed current offset as indicated in Figures 2 and S6. Following this correction and considering H_2_ and H_3_O^+^ as the only reactants involved, the H coverage at a given potential is estimated from the difference of the integrated current and H_2_ flux from the EC-MS signal converted to n_H_ or θ_H_ (using ρ_Cu(111)_) respectively as shown in Figure 2d and summarized in Figure 2e. Implicit cancellation of the pseudocapacitance contribution following a complete voltammetric cycle should lead to closure of the H_ads_ coverage vs potential relationship consistent with total EC-MS collection of H_2_ generated under these conditions. The slow kinetics of recombination hinder such closure for the first voltammetric cycle, although with continuing cycling a closed loop steady-state result is observed in Figure S7.

The proton reduction charge that goes to form H_ads_, prior to MS detection of the onset of H_2_ from the HER, (denoted in Figures 2 and S6 by the two vertical dashed lines) corresponds to a maximum possible H_ads_ coverage of (0.82 ± 0.02) ML regardless of scan rate or cycle number. Comparison of estimated hydride coverage at the HER onset potentials of −0.226, −0.245, −0.260, and −0.290 V for scan rates of 5, 10, 20^,^ and 50 mV s^–1^, respectively, for different scan rates are summarized in Figures S6–S7 and Table S2. The threshold also aligns with the inflection evident between the two voltammetric current peaks that comprise the reduction wave. The subsequent onset of HER is consistent with a further increase in H coverage that might induce the surface-subsurface H phase transition and/or reconstruction of Cu(111) seen in EC-STM experiments wherein the extracted Cu adatoms serve to coordinate and catalyze the hydrogen evolution reaction.^33,34^

Integrating the H_2_ flux from −0.050 V on the positive voltammetric sweep until the background signal is reached again yields between 0.6 nmol cm^–2^ and 0.9 nmol cm^–2^ of H_2_. This corresponds to a conservative estimate of fractional H_ads_ coverage (θ_H_) between 0.36 and 0.61 ML (Figure 2e) depending on the scan rate and negative vertex potential. The integrated H coverage measured for the final voltammetric cycle at 20 and 50 mV s^–1^ is larger than the preceding scans since the potential was held for an extended period at the upper vertex after the voltammetric sweep was finished while collection of the evolved H_2_ continued. The result is congruent with the incomplete decomposition of the hydride phase seen at higher scan rates due to limited recombination kinetics and insufficient time spent at the decomposition potentials. An upper bound on θ_H_ can be estimated by summing the oxidative and H recombination portions for each respective scan rate seen in Figure 2e. The increase in θ_H_ from 0.64 to 0.82 ML with scan rate is evident however the increase also correlates with the shift to more negative vertex potentials where an increase in H_ads_ coverage is predicted by theory.^33^ More negative vertex potentials were used to ensure that the transition between hydride formation and bulk HER was reached when using faster scan rates.

**Figure 3:**
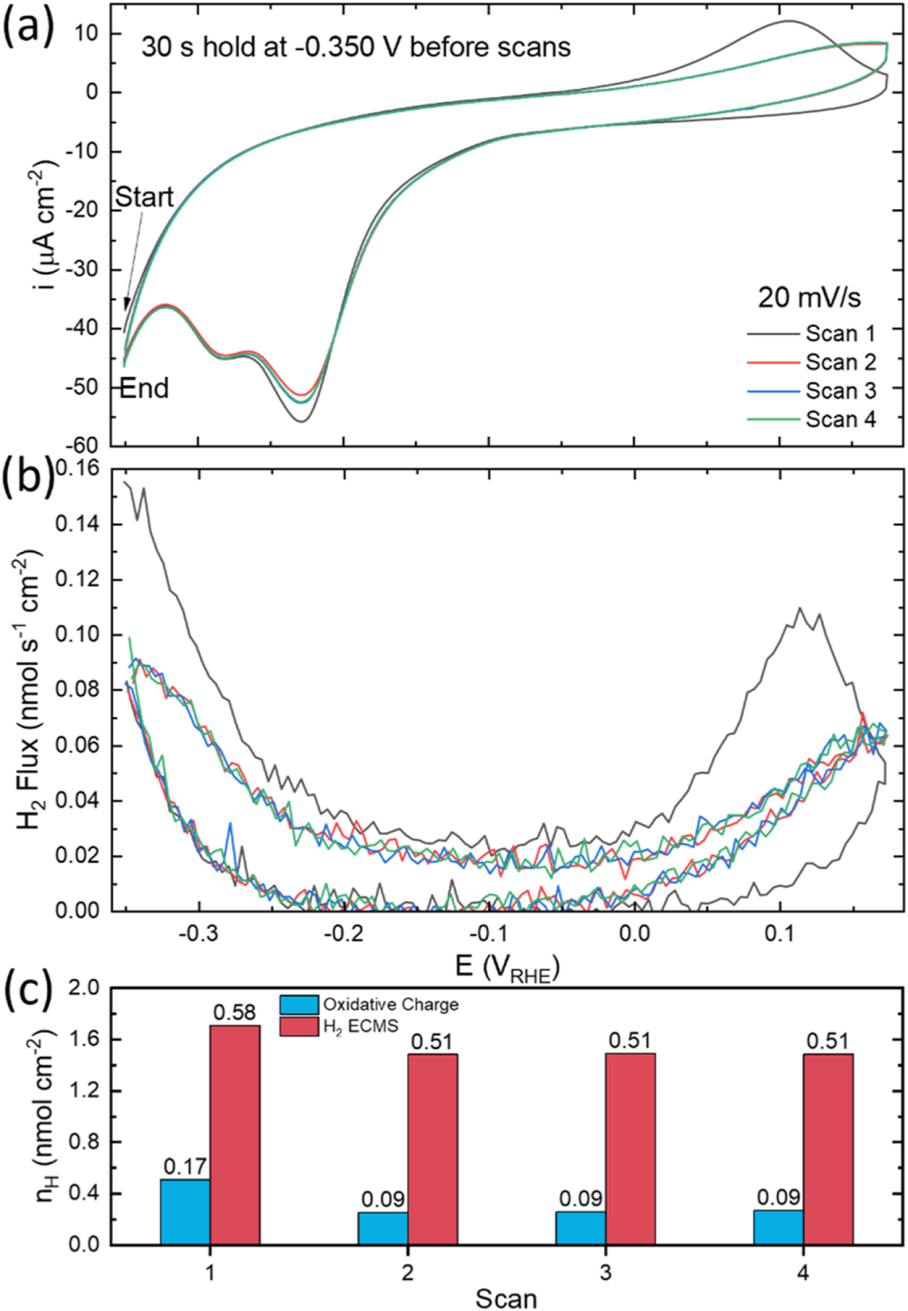
(a) Cyclic voltammetry of Cu(111) in He-saturated 0.1 mol L^–1^ HClO_4_ and the accompanying (b) H_2_ flux measured by EC-MS following an initial 30 s pretreatment at −0.350 V. The H_2_ flux and oxidative peak on the anodic sweep were (c) integrated and converted to n_H_ (nmol cm^–2^) and are labeled above the bar graph in terms of the H_ads_ fractional surface coverage θ_H_.

The impact of extended polarization at negative potentials where the hydride is formed was examined by voltammetry where the initial potential was held at −0.325 and −0.35 V for 30 s, respectively. As shown in Figure 3 the voltammetric kinetics of hydride decomposition following extended polarization at negative potentials are substantially faster than seen for voltammetry initiated at positive potentials on a hydride-free surface, i.e., Figures 1 and 2. Following pretreatment at −0.325 V, Figure S8, the peak potentials for the first oxidative wave and EC-MS hydride decomposition are downshifted to 0.126 V (≈34 μC cm^–2^ or ≈0.35 nmol cm^–2^) and ≈0.135 V (1.59 nmol cm^–2^), respectively. For pretreatment at a slightly more negative potential of −0.35 V, the respective peaks in Figure 3 are downshifted further to 0.107 V (≈28 μC cm^–2^ or ≈0.29 nmol cm^–2^) and ≈0.118 V (1.29 nmol cm^–2^), respectively. For both sets of experiments, the subsequent voltammograms with continuous scanning yield the same positive shift in peak positions to ≈0.165 and ≈0.175 V, respectively. If the oxidative charge is ascribed solely to H_ads_ oxidation to hydronium while the H_2_ flux is assigned to H_ads_ desorption by recombination the summation gives a fractional H_ads_ coverage of 0.66 ML at −0.325 V and 0.75 ML at −0.350 V, subject to the scan rate used. These values are well aligned with the predicted potential-dependent coverage from theoretical calculations detailed earlier.^33^ Accordingly, excursions to more negative values lead to higher H_ads_ coverages and H-induced reconstruction of the Cu(111) that collectively impact the onset and acceleration of the HER. For subsequent cycles, the maximum H_ads_ coverage evaluated from the voltammograms decreased to (0.6 ± 0.01) ML, Figure 3, and (0.54 ± 0.015 ML), Figure S8, respectively, which reflect kinetic hindrance in hydride formation during voltammetric cycling. At more negative potentials where the coverage exceeds 0.75 ML, theory predicts a reconstruction of the existing (4 × 4) structure with the formation of adatoms, dimers, and related vacancy structures that maintain the (4 × 4) periodicity.^33^ The adatoms and vacancies facilitate both higher H_ads_ coverage and enhanced reaction kinetics. Recent computational work indicates that adsorbed H is required and works synergistically with CO to create sites of CO-bound Cu atoms that sit 0.1 nm above the top surface accounting for surface reconstruction that accompanies CO_2_RR.^39^ The increased H_ads_ coverage and enhanced hydride decomposition and oxidation characteristics following aging, Figures 3 and S8, and/or during excursions to more negative potentials, <−0.325 V, Figure 2, are consistent with this picture.

### Chronoamperometric EC-MS Measurements in 0.1 mol L^–1^ HClO_4_

**Figure 4:**
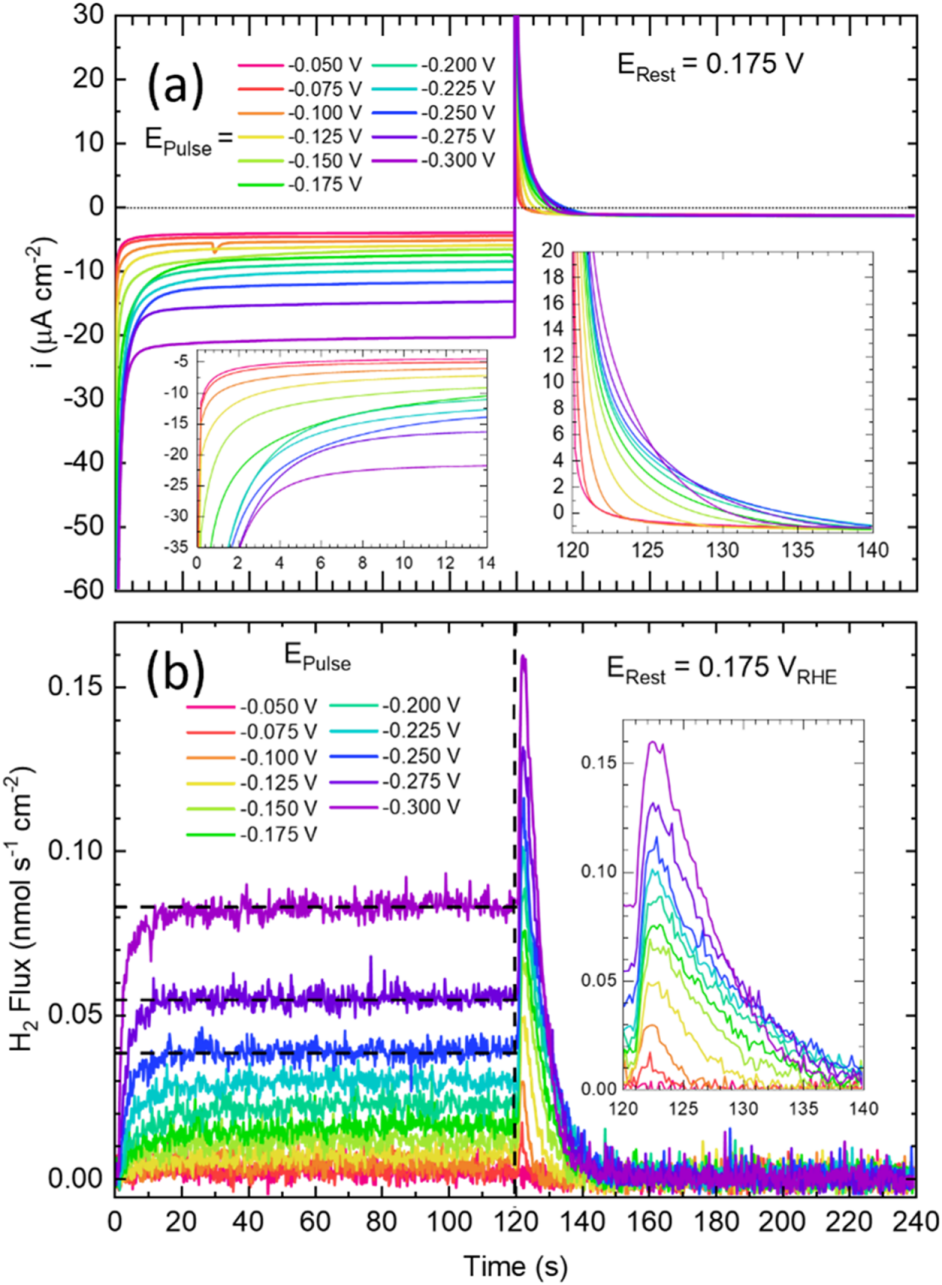
(a) Chronoamperometry and (b) H_2_ flux during potential pulse measurements on Cu(111) in He-saturated 0.1 mol L^–1^ HClO_4_. The horizontal dotted line in (a) indicates zero current. The horizontal dashed lines in the H_2_ flux plot indicate the steady-state HER (HER_SS_) flux at the three most negative *E*_Pulse_ as determined by averaging the last 60 s of the flux during *E*_Pulse_. The vertical dashed line indicates the transition to *E*_Rest_. The insets in (a) magnify the transient components of current for both E_Pulse_ a E_Rest_ and the inset in (b) magnifies the transient period at *E*_Rest_ associated with the decomposition of hydride to H_2_ via H_ads_ recombination.

Potential pulse EC-MS measurements were used for a more complete quantitative analysis of the transient and steady-state behavior associated with surface hydride formation and decomposition.^36^ The experiment involved stepping the potential from an initial rest state, *E*_Rest_ = 0.175 V to a more negative potential, *E*_Pulse_ for 2 min and then stepping back to *E*_Rest_ for 2 min. The *E*_Pulse_ value was then progressively advanced in −25 mV increments (Figures 4 and S1). The chronoamperometric experiment mitigates convolution of the H_2_ diffusional lag from the dynamic potential scan used in voltammetry. Sequential chronoamperometric transients for each pulse cycle between *E*_Pulse_ and *E*_Rest_ in He-saturated 0.1 mol L^–1^ HClO_4_ electrolyte are superimposed in Figure 4a. Starting from *E*_Pulse_ of −0.05 V, the steady-state current densities at *E*_Pulse_ and *E*_Rest_ remain roughly < −5 μA cm^–2^ and ≈ −2 μA cm^–2^, respectively. These negative currents arise from the oxygen reduction reaction (ORR) due to trace (on the order of pmol L^–1^ s^–1^) O_2_ flux, possibly from leakage through various joints and connections of the EC-MS cell, as no change in H_2_ flux was detected via the mass spectrometer (Figure 4b).^36^ The current density when E_Pulse_ = -0.1 V increases to ≈ −5.5 μA cm^–2^, consistent with an increase in the steady-state HER rate (Figure S9). Stepping to −0.150 V increases the steady-state HER rate modestly, although a notable change in the first ≈25 s of the current transient is observed (Figures 4b and S9). Compared to −0.150 V, the time to reach a steady-state current increases at −0.175 and −0.200 V before decreasing for *E*_Pulse_ ≥ −0.225 V. The slower time constant for the −0.150 and −0.225 V transients is associated with the nucleation and growth of the surface hydride phase superimposed on proton reduction to H_2_ and double-layer charging. An additional contribution from anion desorption is possible although this is believed to be unlikely for perchlorate electrolytes in contrast to the measurable influence of sulfate, phosphate, or chloride ions.^36^ Pulses in the 100 μmol L^–1^ HCl + 0.1 mol L^–1^ HClO_4_ electrolytes show similar behavior albeit shifted negatively ≈ −75 mV due to inhibition of hydride formation by the adsorbed chloride (Figure S10). This results in a more convolved current transient (left inset of Figure S10a) that involves the dynamics and additional charge necessarily associated with the sequence of Cl^–^ desorption and the formation of hydride. Comparing the transient charge at a pulse potential of −0.3 V (−288 vs −253 μC cm^–2^ for Cl vs HClO_4_, vide infra) reveals −35 μC cm^–2^ of additional charge due to Cl^–^ desorption. Likewise, hydride formation in 0.1 mol L^–1^ H_2_SO_4_ involved a transient charge of −275 μC cm^–2^ where the excess charge relative to 0.1 mol L^–1^ HClO_4_ is attributed to sulfate desorption (Figure 5a in ref (36)).

Upon returning to *E*_Rest_ an oxidative transient current is observed (Figure 4a right inset), with the duration and charge increasing as the potential is stepped from more negative *E*_Pulse_ values between −0.05 to −0.225 V. The oxidative charge reflects a combination of double-layer charging, anion adsorption, and/or hydride decomposition by oxidation to hydronium. As noted above, although anion adsorption on Cu surfaces is well established for sulfate or halide electrolytes, evidence for specific perchlorate anion adsorption is at best ambiguous. Significantly, a recent STM study of a water-dosed Cu(111) surface in cryogenic vacuum environment revealed ordered adlayers that were ascribed to water and hydronium structures, a subset of which were similar to those observed in ECSTM studies.^40,46−48^ Inspection of the oxidative current transients at *E*_Rest_ reveals that the time to reach the steady-state background current increases from 5 to 20 s as *E*_pulse_ is made more negative. However, when *E*_Pulse_ < −0.225 V an inversion and decrease in the time constant is evident with the transient lifetime dropping to 15 s following polarization at *E*_Pulse_ of −0.3 V. The acceleration of the oxidative process (in 0.1 mol L^–1^ HClO_4_) following more negative pulse potentials is consistent with that seen in the related voltammetric experiments, Figures 3 and S8. One possible explanation is that Cu adatom formation driven by higher hydride coverages formed at more negative potentials leads to accelerated hydride decomposition with the step back to the rest potential.

Interestingly, the accelerated hydride decomposition seen in 0.1 mol L^–1^ HClO_4_ is not evident following titration with 100 μmol L^–1^ Cl^–^, nor is it evident in prior sulfuric acid experiments. In the presence of Cl^–^ the oxidative current transient increases abruptly when *E*_pulse_ is −0.2 V and effectively saturates for *E*_pulse_ < −0.225 V consistent with completion of the hydride phase over a small potential window (Figure S10a and right inset). Also of interest, the oxidative current transients in the presence of Cl^–^ (Figure S10b) do not accelerate when stepping from more negative potentials, e.g., −0.3 V, in contrast to that observed in pure 0.1 mol L^–1^ HClO_4_. This might be attributed to anion-facilitated quenching of the elevated adatom population associated with the hydride phase formed at more negative potentials.

Following 120 s of polarization at *E*_Pulse_ the potential is stepped back to *E*_Rest_ and the increase in H_2_ flux provides an unambiguous signature of hydride formation by virtue of its decomposition via H recombination to H_2_ at *E*_Rest_ (Figure 4b and right inset). Notably, this occurs well positive of 0.0 V. The hydride decomposition peak at *E*_Rest_ continues to grow as *E*_Pulse_ is stepped more negative with the integrated H_2_ flux as a function of *E*_Pulse_ shown in Figure S11. An inflection, evident near −0.225 V, is suggestive of a plateau or phase boundary in the H_ads_ coverage. Hydride formation at *E*_Pulse_ overlaps the onset and development of HER with the latter reaching a steady-state rate within the first 30 s of the 120 s dwell period. This decay rate is accelerated substantially in the presence of Cl^–^ with its strong absorption stimulating H_ads_ recombination to occur in nearly half of the time (10 s) for all pulse potentials (Figure S10b). This observation also supports the attribution of the sluggish decay curve, in the absence of halide, to the slow decomposition kinetics of hydride rather than H_2_ diffusion across the thin layer of the electrolyte. In pure 0.1 mol L^–1^ HClO_4_ and *E*_Pulse_ < −0.2 V, the additional increase in peak height upon stepping to *E*_Rest_ partly reflects an increase in H_ads_ coverage but is convolved with residual H_2_ diffusing across the electrolyte following steady-state HER at *E*_Pulse_ (Figure S11). The decay associated with this residual H_2_ significantly affects the shape of the hydride envelope. Quantitative analysis is further complicated by the competitive, first-order oxidation of H_ads_ to H_3_O^+^. Nonetheless, the more rapid decay in H_2_ flux following polarization at *E*_Pulse_ values < −0.275 V speaks to an increase in the kinetics for H_ads_ recombination and/or the H_ads_ oxidation reaction. The acceleration is most likely related to H-induced reconstruction of the surface that results in more reactive low-coordinate Cu adatoms at higher H_ads_ coverages.^33^

### Quantitative Analysis of Hydride Coverage

**Figure 5:**
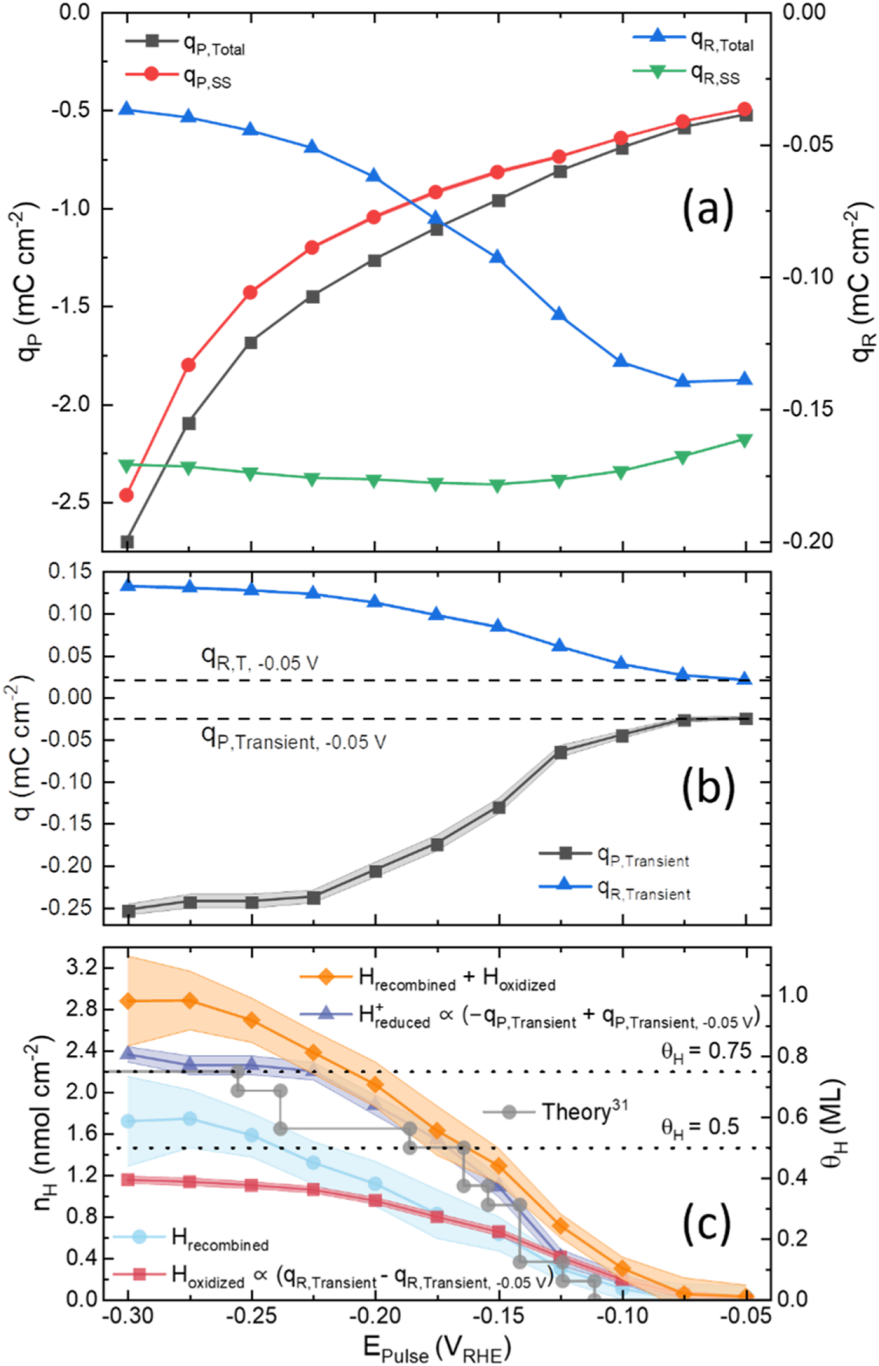
(a) Total (q_P,Total_ and q_R,Total_) and steady-state (q_P,SS_ and q_R,SS_) chronoamperometric charge and (b) their difference, the transient charge (q_P,Transient_ and q_R,Transient_), for *E*_Pulse_ and *E*_Rest_ during negative step potential pulse measurements in 0.1 mol L^–1^ HClO_4_ (Figure 4). (c) Total H coverage determined from either the background corrected hydronium reduction wave, H_reduced_^+^, or the sum of hydride recombination, H_recombined_, and H_ads_ oxidation to hydronium, H_oxidized_. The transparent background for each curve represents the standard deviation. Also plotted is the coverage (denoted as “theory” in the legend) predicted by computational simulations from Cheng et al. for pH 1 electrolyte.^33^

The hydride coverage can be quantified using the steady-state approximation for the HER, as done previously in similar experiments with sulfuric acid electrolyte.^36^ In contrast to experiments in sulfate- or chloride-containing media, chemisorption of perchlorate is negligible with no order anion layers reported in the plurality of ECSTM studies therefore, no charge will be assigned to anion adsorption.^40,47^ For chronoamperometry, separating the steady-state charge (q_P,SS_, q_R,SS_) from the total measured charge (q_P,Total_, q_R,Total_) for both *E*_Pulse_ and *E*_Rest_ (Figure 4a,b), isolates the transient charges (q_P,Transient_, q_R,Transient_) associated with reductive hydride formation at *E*_Pulse_ and its subsequent oxidation at *E*_Rest_, respectively. The steady-state HER approximation is evaluated by averaging the current over the final 60 s of *E*_pulse_ or *E*_rest_ and multiplying by the total pulse or rest time (120 s each) to obtain the charge (q_P,SS_, q_R,SS_). As shown in Figure 5, the approximated steady-state charge during *E*_Pulse_ or *E*_Rest_ is subtracted from the total measured charge to yield the transient charge, q_P, Transient_ or q_R,Transient_, respectively. Initially, for *E*_Pulse_ ≥ −0.175 V, the transient charges for *E*_Pulse_ and *E*_Rest_ are approximately equal to opposite polarity, congruent with reversible capacitive charging of the interface. The onset of hydride formation introduces asymmetry in the net transient charge, as captured in Figure 5b. Specifically, |q_P,Transient_/q_R,Transient_| increases from around 1 at *E*_Pulse_ = −0.05 V (prehydride formation) to a plateau of ≈2 at −0.225 V, due to surface hydride formation. This charge analysis ignores the contribution of double-layer capacitance associated with stepping between *E*_Pulse_ to *E*_Rest_. However, negligible hydride formation occurs prior to −0.05 V and thus an upper bound for the corresponding net change associated with hydride formation is evaluated based on the difference between the transient charge at −0.05 V (hydride-free) and −0.225 V (hydride-covered), giving (−218 ± 8) μC cm^–2^ of charge to reduce H^+^ to surface hydride (Figure 5c). Normalizing this charge to θ_H_ on Cu(111) yields a saturated coverage of (0.77 ± 0.03) ML. Assuming that the decomposition of surface hydride is a competitive process between oxidation and recombination reactions, the contribution of oxidation can be determined by measuring the background corrected oxidation charge when stepping from *E*_Pulse_ to *E*_Rest_. At its maximum, this yields Δq_R,Transient_ (103 ± 2) μC cm^–2^ (Figure 5b) that corresponds to a loss of (0.36 ± 0.01) ML of H (Figure 5c). The contribution from the H recombination process is evaluated directly from the 2 amu EC-MS measurement.

Analysis of the H_2_ flux also involves partitioning the measured H_2_ (H_2,Total_) into contributions from the HER (H_2,HER_) and hydride decomposition by H recombination (H_2,Hydride_) (Figure S12). Previous work utilized the total collection capacity of the EC-MS platform^41^ to deconvolve these terms. It was assumed that steady-state HER at *E*_pulse_ is rapidly (instantly) attained, with the EC-MS detection delayed due to H_2_ diffusion across the thin electrolyte layer.^36^ The EC-MS measurement is particularly effective in revealing the onset of hydride formation as the HER at *E*_rest_ drops rapidly to zero while the H_ads_ recombination to yield H_2_ takes several seconds, Figures 4b, S9, and S11. The integrated result of the small H_2_ quantity detected, following polarization at −75 mV, is consistent with assigning the transient charge at −0.05 V to the capacitance, Figure 5b. Integration of the decomposition peak upon stepping to *E*_Rest_ shows a monotonic relationship between hydride coverage and *E*_Pulse_ between −0.10 to −0.25 V, Figure 5c. A plateau is evident between −0.25 and −0.3 V, corresponding to a hydride coverage of (0.54 ± 0.05) ML. This is lower than the 0.67 ML observed in 0.1 mol L^–1^ H_2_SO_4_ using the same procedures which reflects the role of sulfate adsorption in spurring the recombination reaction.^36^

Deconvolving the charge contributions during negative potential pulses in 100 μmol L^–1^ HCl + 0.1 mol L^–1^ HClO_4_ (Figure S13) requires consideration of Cl^–^ desorption during hydride formation and Cl^–^ adsorption during hydride decomposition. As noted earlier, the charge associated with hydride formation in the presence of Cl^–^ exceeds that for neat perchloric acid by ≈35 μC cm^–2^. Studies of Cl^–^ adsorption on Ag(111) indicate an electrosorption valancy of 0.44 and based on the similarity between the pzc of Cu and Ag this value is adopted.^49^ Accordingly, the excess charge of ≈35 μC cm^–2^ corresponds to a fractional Cl^–^ coverage θ_Cl_ of 0.28 ML compared to 0.33 ML expected for a (√3 × √3)*R*30° overlayer structure that can be taken as a first-order estimate based on reported adlayer surface structures.^44,50^ Alternatively, an inverse analysis using the electrosorption normalized charge for the (√3 × √3)*R*30° adlayer of ≈41 μC cm^–2^ can be used to provide a charge-based estimate of the hydride coverage at −0.35 V, θ_H_ of 0.89, not surprising since double-layer charge was not considered. It should also be noted that the coexistence of H and Cl adlayer structures proposed in recent theory might lead to variation in the electrosorption valancy that will need to be considered.^51^ Meanwhile the θ_H_ determined by integration of H_2_ flux during desorption in the presence of Cl^–^ appears to converge to 0.75 ML (Figure S13c), while that for perchloric acid does not exceed 0.6 ML, supporting the idea that Cl adsorption favors H recombination over oxidation.

In the absence of anion adsorption, the sum of the oxidative and recombination components associated with hydride decomposition measured at *E*_Rest_ can be compared with the proton reduction charge used to form the hydride (Figure 5c). Good agreement between the metrics is achieved for ≥ −0.225 V where ≈0.75 ML of H_ads_ is expected. This is further corroborated by the 0.75 ML of H measured through recombination when 100 μmol L^–1^ HCl is present in 0.1 mol L^–1^ HClO_4_. At more negative potentials, the transient charge at *E*_Pulse_ plateaus, whereas the sum of oxidation and recombination increases slightly to ≈1 ML by −0.3 V. However, this is accompanied by an increase in standard deviation that weakens the significance of the increase. Recent computational studies of H adsorption on Cu(111) indicate two domains of hydride stability with a coverage of 0.56 and 0.75 ML that correspond to slightly different (4 × 4) periodic structures similar to that observed by ECSTM.^33^ The predicted coverage (Figure 5c, “Theory”) overlaps the present and previous EC-MS studies in perchloric and sulfuric acid. The chief distinction between sulfuric acid, hydrochloric acid, and perchloric acid is the strength of anion adsorption; specifically, sulfate and/or chloride adsorption favors hydride decomposition by recombination, whereas the absence of perchlorate adsorption results in a measurable increase in the amount of H_ads_ removed by oxidation.

### Impact of *E*_Rest_ on Hydride Decomposition 0.1 mol L^–1^ HClO_4_

**Figure 6:**
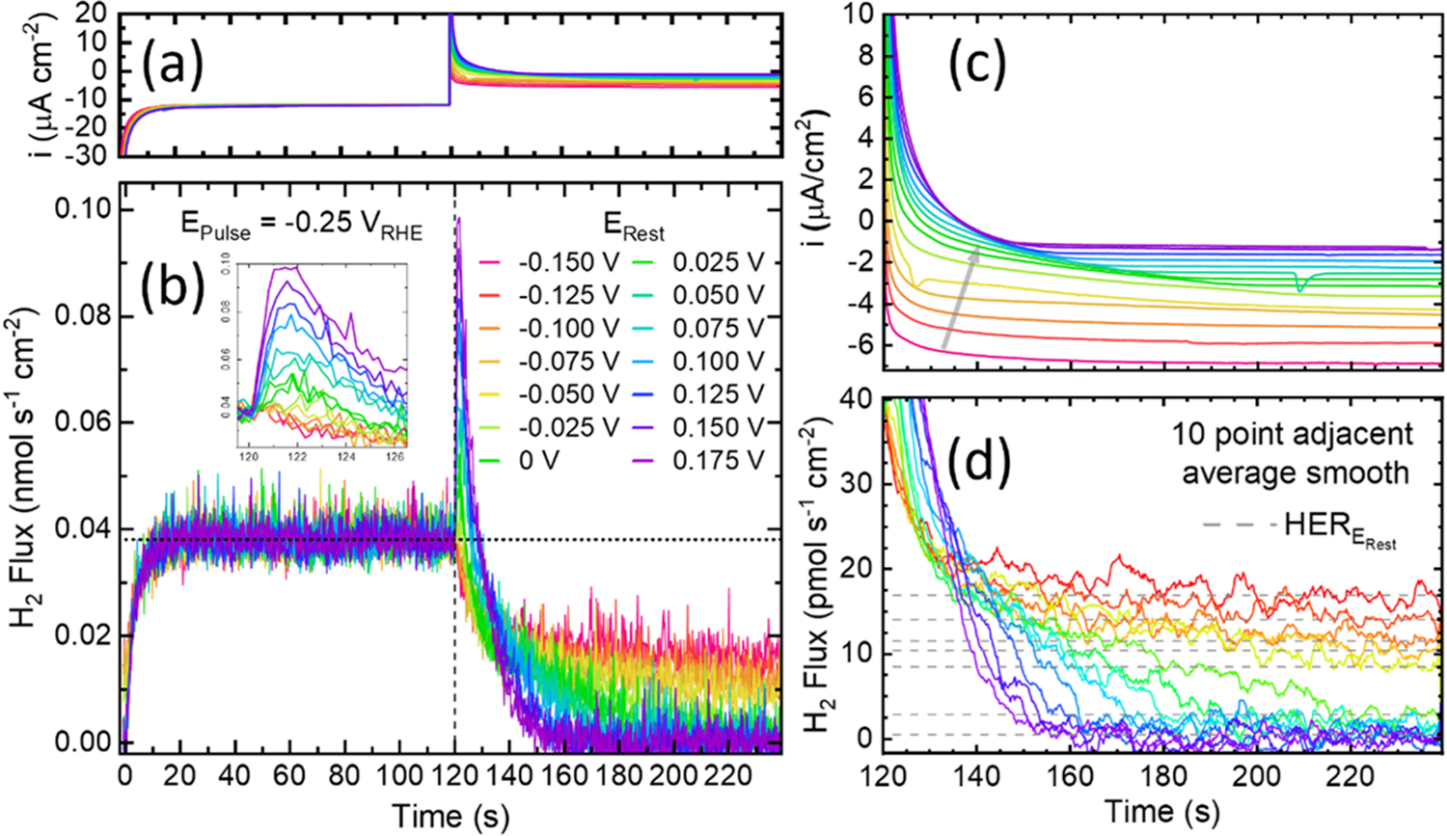
(a, c) Chronoamperometry and (b, d) H_2_ flux during variable *E*_Rest_ measurements on Cu(111) in 0.1 mol L^–1^ HClO_4_. The inset in (b) magnifies the 2 amu hydride decomposition peak, while the magnified current and H_2_ flux profile for the entire *E*_Rest_ cycle are presented in (c) and (d), respectively. The horizontal dashed line in (b) represents the average steady-state H_2_ flux during the *E*_Pulse_. The gray dashed lines in (d) reflect the steady-state HER rate at *E*_Rest_ found by averaging the last 5 s of the H_2_ flux for each *E*_Rest_.

**Figure 7:**
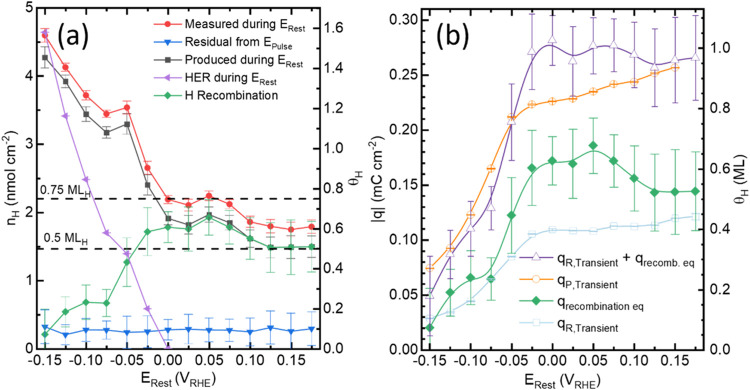
(a) Total and partitioned quantities of n_H_ determined via the EC-MS and (b) charge analysis from (Figure 6a) as a function of *E*_Rest_. The q_recombination equivalent (eq)_ (green diamonds) in (b) is the hydride charge equivalent that is not realized due to the loss of H_2_ by recombination, as measured by EC-MS (a). The error bars represent the standard deviation of the measurement.

To further explore hydride decomposition and its partitioning between H_ads_ recombination to H_2_ versus oxidation to hydronium, a similar set of pulse experiments were performed where *E*_rest_ was changed as *E*_Pulse_ was held constant (Figure 6). For this experiment, *E*_Pulse_ was chosen to be −0.25 V, which corresponds to the well-defined plateau in hydride coverage established in the previous experiment. The detailed potential program is shown in Figure S2 with the substrate initially poised at 0.175 V, where the surface is known to be hydride-free. The chronoamperometric, Figure 6a, and H_2_ flux, Figure 6b, measurements during repetitive stepping to *E*_Pulse_ of −0.25 V demonstrate good reproducibility while the choice of *E*_Rest_ significantly impacts the rate and nature of hydride decomposition. For the first *E*_Rest_ value of −0.15 V both current and H_2_ flux decay to a new steady-state HER value (Figure 7a “HER During *E*_Rest_”). As *E*_Rest_ is sequentially increased in +25 mV increments, up to −0.025 V the current and H_2_ flux transients become more sluggish. Once *E*_Rest_ > −0.025 V a clear uptick in H_2_ flux (inset shown in Figure 6b) from hydride decomposition by recombination accompanies the step from *E*_Pulse_ to *E*_Rest_. At the same time a slow transient oxidative process is observed in Figure 6c that reflects the tail of double-layer charging and H_ads_ oxidation to hydronium relaxes, decaying over ≈80 s at −0.025 V (compared to ≈30 s at 0.175 V), to a steady-state reduction current associated with residual oxygen.

Advancing *E*_Rest_ further leads to acceleration of hydride decomposition with the H_2_ flux reaching the 0 nmol s^–1^ cm^–2^ baseline well within the 120 s duration for all *E*_Rest_ > −0.025 V. The 0 nmol s^–1^ cm^–2^ baseline is helpful in quantifying the HER kinetics that are otherwise obscured by the residual oxygen reduction reaction. Indeed, the H_2_ flux approaches zero at 0.0 V, consistent with this idea. Direct comparison between the current density and H_2_ flux at the end of *E*_Rest_ is shown in Figure S14 consistent with a residual oxygen reduction reaction current that increases from 1.5 μA/cm^2^ at 0.175 V to reach a transport-limited value of ≈3.0 μA/cm^2^ near 0.0 V. Integration of the *E*_Pulse_ current transient at −0.250 V following the initial step from an *E*_Rest_ of 0.175 V and applying the steady-state HER approximation yields 0.246 mC cm^–2^ of transient charge, in good agreement with the early negative step potential pulse measurements (Figure 5b). This charge equates to a H_ads_ coverage of 0.78 ML based on the strategy used to determine H_reduced_^+^ in Figure 5c.

As in earlier EC-MS analyses, the steady-state and total collection approximation is used to account for the different sources of H_2_ measured during *E*_Rest_. This includes not only H_ads_ desorption via recombination but also ongoing HER at *E*_Rest_, along with the collection of H_2_ produced during *E*_Pulse_ but measured during *E*_Rest_ due to diffusional lag. The integrated H_2_ flux over the duration of E_Rest_ is given as “*Measured during E*_*Rest*_” in Figure 7a. The contribution of H_2_, due to diffusional lag from the HER at *E*_Pulse_, “*Residual from E*_*Pulse*_,” is estimated by subtracting the total H_2_ measured during *E*_Pulse_ from the product of the steady-state H_2_ flux and pulse time at *E*_Pulse_ (Figure S15). This “*Residual from E*_*Pulse*_,” is then subtracted from the “*Measured during E*_*Rest*_” to leave the contribution “*Produced during E*_*Rest*_” attributed to H recombination and HER as summarized in Figure 7a. Separation of H_2_ generated from H_ads_ recombination versus HER at *E*_Rest_ can be approximated by assuming that the last 5 s of the EC-MS transient is representative of a steady-state HER flux (Figures 6d and S14) that multiplied by t_Rest_ gives the contribution “*HER during E*_*Rest*_” shown in Figure 7a. The approach works well, although close inspection of the *E*_Rest_ transients between −0.1 and −0.025 V indicates the slow rate of H recombination may overlap with the 5 s averaging evaluation of the HER contribution (Figure 6d). With that qualification, subtraction of “*HER during E*_*Rest*_” from “*Produced during E*_*Rest*_” yields the amount of H_2_ generated by hydride decomposition via the recombination pathway as indicated by *“H Recombination”* in Figure 7a.

The analysis indicates a near monotonic dependence of H_ads_ desorption by recombination as *E*_Rest_ is increased from −0.15 to −0.025 V. As *E*_Rest_ is increased further from −0.025 and 0.075 V, the amount of H_ads_ desorbed by recombination rises to reach a maximum near 0.6 ML before settling to a value near 0.5 ML for *E*_Rest_ > 0.125. As the total amount of hydride to be decomposed is fixed, the transition or decrease in the amount desorbed by recombination is attributed to the onset of the oxidative removal of H_ads_ as hydronium. This is consistent with *E*_Rest_ becoming greater than 0 V. Interestingly the transition also overlaps with the potential range where two minor peaks develop during cyclic voltammetric experiments as shown in Figure 1. The kinetic limitations on hydride decomposition are such that in voltammetric experiments the peak reaction is observed at much more positive potentials. For chronoamperometry, the potential and the time dependence are well captured by the H_2_ signal (Figure 6d) where hydride decomposition takes ≈100 s at −0.05 V but only requires 40 s at 0.075 V. The trend continues at higher *E*_Rest_ with the transition taking only ≈20 s at 0.175 V which is convolved with the time required for H_2_ to diffuse across the electrolyte to the MS entry port.

The charge transients (q_P,Transient_ and q_R,Transient_) for the positive step potential pulse measurements (Figure 7b) were determined by following the same strategy used for the negative step potential pulse measurements, where the steady-state charge was subtracted from the total charge (Figure S16) for both *E*_Pulse_ and *E*_Rest_. The steady-state currents were determined by averaging the current for the final 3 s of *E*_Pulse_ and *E*_Rest_, respectively, and the charge was found by multiplying by the time of the pulse or rest (120 s each). As with the EC-MS analysis this assumes the steady-state processes are not influenced by transient events at the electrode surface. This may be a less accurate procedure for assessing the current compared to H_2_ flux, given that other phenomena may contribute such as parasitic ORR, double-layer, and/or adsorption-related pseudocapacitance. The experiment begins by first forming a hydride at −0.250 V that yields a q_P,Transient_ of −0.246 μC/cm^2^ (not indicated in Figure 7b). For the first positive E_Rest_ step to −0.15 V the q_R,Transient_ is only 30 μC cm^–2^. This increases to nearly 110 μC cm^–2^ for an *E*_Rest_ of −0.025 V with only modest increases thereafter. The q_P,Transient_ associated with the next *E*_Pulse_ corresponds to reforming the increment of hydride decomposed on the previous *E*_Rest_ step. Assuming the reformed hydride phase after each *E*_Pulse_ is the same, the difference between q_P,Transient_ (e.g., the charge for hydride formation) and its subsequent oxidative decomposition must equal the amount of hydride decomposed through the recombination to H_2_. The materials balance is examined by converting n_H_ from recombination (“H Recombination” Figure 7a) to its charge equivalent, q_recombined equivalent_. Combining this with q_R,Transient_ for hydride decomposition by oxidation to hydronium enables a comparison to the hydride formation charge, q_P,Transient_. Assuming the hydride structure is conserved over multiple formation-decomposition cycles the sum of the two decomposition pathways should equal the charge needed to reform the hydride. The combination shows that complete decomposition of the hydride phase occurs at potentials > −0.05 V. Reasonable agreement between the charge and H_2_ flux measurements is encouraging especially considering the uncertainties in the charge analysis associated with residual oxygen reduction reaction, double-layer capacitance, and the assumption implicit in the steady-state analysis. Future exploration will focus on rectifying observed differences between chronoamperometry and EC-MS during potential pulse measurements. Of particular interest is the impact of anion adsorption on the partitioning of the decomposition reaction between oxidative desorption versus H recombination. Previous work with sulfuric acid indicated that strong anion chemisorption accelerates the rate of the recombination pathway, such that desorption is complete before the overpotential increases enough for the oxidative desorption channel to become significant. Conversely, the decrease in H recombination for E_Rest_ > 0.05 V (Figure 7) points to a potential dependent increase in hydride oxidation.

Looking beyond hydride formation and decomposition to its effect on hydrogenation reactions, two distinctive regimes of behavior may emerge. In the first instance formation of the hydride overlaps the potential region where many hydrogenation reactions occur and the hydride phase itself may serve as the catalytic template but perhaps also as an active reaction.^22,23^ A prime example for consideration is the reduction of oxygen (ORR) where product selectivity between hydrogen peroxide versus water is known to be a sensitive function of anion adsorption and surface structure.^52,53^ Indeed, the transitions in ORR product selectivity directly overlap the potential regime where hydride formation and decomposition occur motivating the need for further study.^35,36,52,53^ A second regime of behavior to consider involves cross-coupling between potential-induced hydride formation and its desorption with the hydrogenation reaction of interest. An example of this is CO_2_/CO reduction on Cu surfaces using pulsed potential, or pulsed current, schemes where the perturbation repeatedly transits the hydride formation and decomposition window.^17,54^ In this instance the binding energy of adsorbed hydrogen varies greatly during the process with hydride first being formed on the negative-going potential pulse while during the reverse step adsorbed hydrogen is available for further hydrogenation reactions but at a much lower energy cost.^54^

## Conclusions

Surface hydride formation on Cu(111) in perchloric acid is associated with two reductive waves. Chronoamperometry measurements support theoretical predictions of a domain of uniform hydride coverage near 0.75 ML exists between −0.225 and −0.3 V.^33^ The inflection between the two voltammetric reduction waves is associated with reaching ≈0.8 ML of hydride followed by the onset of the HER. The latter is also congruent with computational predictions detailing the formation of low coordination sites that may be responsible for the increase in HER kinetics.^33^ Variation of the scan rate in cyclic voltammetry reveals two additional minor peaks centered near −50 mV likely associated with the destabilization/stabilization of the hydride phase. Above −0.050 V the surface hydride undergoes decomposition either by H_ads_ recombination to H_2_ or by oxidation back to H_3_O^+^, the rate of the latter increasing with potential. Kinetic limitations of the oxidation reaction manifest in the displacement of the oxidation wave to higher potentials for higher voltammetric scan rates. This is also evident in chronoamperometry where compression of the q_R_ time constants occurred as *E*_Rest_ increased from −0.025 to 0.175 V while the net oxidative charge was largely independent of the applied potential. Exposure to more negative potentials for longer times accelerates subsequent voltammetric hydride decomposition kinetics, presumably due to structural rearrangement and/or elevated H_ads_ coverage. The overpotential required for hydride formation on Cu(111) may impact the onset of hydrogenation reactions. Likewise, the slow decomposition kinetics for hydride decomposition and the prospect of weakly bound H_ads_ being available at more positive potentials have implications for the use of pulse potential activation of hydrogenation reactions.

## Supporting Information Available

The supporting information contains; continuous plots of the negative and positive potential pulse experiments, electrochemical impedance spectroscopy, cyclic voltammetry (CV) where scan rate was modulated without pausing, CVs with varying concentrations of HCl present in the electrolyte , versions of Figure 2 for additional scan rates, a table tabulating values from Figure 2 and Figure S6, plots of maximum H coverage vs potential during CVs at different scan rates, CVs with negative potential holds prior to the first scan and corresponding H coverage, plots that zoom-in on current and H_2_ flux during potential pulses, negative potential pulse experiments with HCl in the electrolyte, plots of integrated H_2_ flux during *E*_Rest_ of negative step potential pulse measurements, quantities of n_H_ measured by the EC-MS during negative potential pulse measurements, charges from negative potential pulse measurements, steady-state current density from the last 5 s of E_Rest_ during potential pulse measurements, total and steady-state H measured during E_Pulse_ for positive step potential pulse measurements, and total or steady-state charges from *E*_Pulse_ during positive step potential pulse measurements (PDF)

## Footnotes

* Certain commercial equipment, instruments, or materials are identified in this paper to specify the experimental procedure adequately. Such identification is not intended to imply recommendation or endorsement by the National Institute of Standards and Technology, nor is it intended to imply that the materials or equipment identified are necessarily the best available for the purpose.
