# Supplementary material for: Hydride Formation and Decomposition on Cu(111) in HClO4
Source: J Am Chem Soc. 2025 Jan 27;147(5):4038–51. doi: 10.1021/jacs.4c12782 (PMC11844892; doi:10.1021/jacs.4c12782)
Supplement: Supplementary file 1 — ja4c12782_si_001.pdf [file ja4c12782_si_001.pdf]

Supporting Information for

Hydride Formation and Decomposition on Cu(111) in $HClO_4$

David Raciti,[1] and Thomas P. Moffat*,[1]*

[1]Material Science and Engineering Division, National Institute of Standards and Technology, 100 Bureau Drive, Gaithersburg, MD 20899, USA

Official contribution of the National Institute of Standards and Technology; not subject to copyright in the United States.

ORCID:

David Raciti: 0000-0002-9580-4524

Thomas P. Moffat: 0000-0003-4377-1692

*Corresponding Authors: David Raciti (david.raciti@nist.gov), Thomas P. Moffat (thomas.moffat@nist.gov)

Supporting Figures

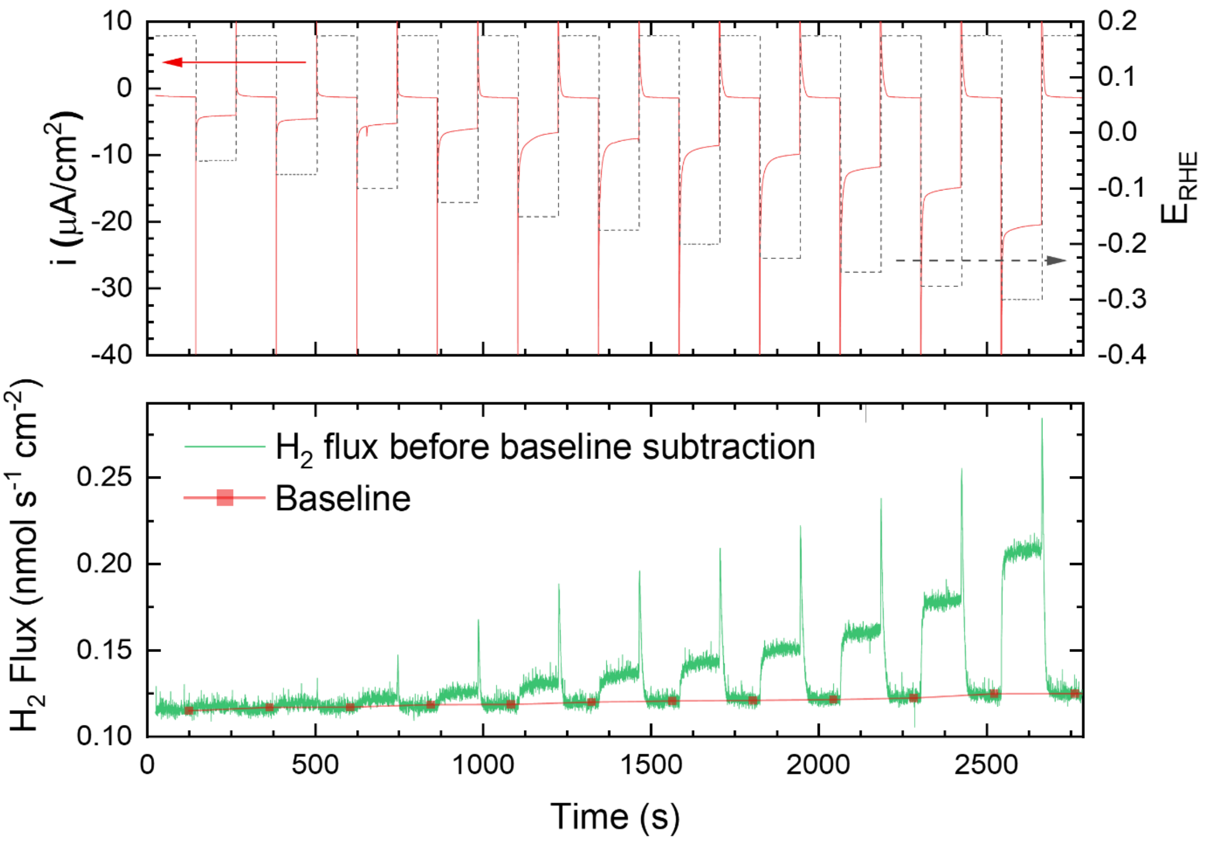

Figure S1. Negative step potential pulse measurements on Cu(111) in He saturated 0.1 mol L^{-1} $HClO_4$. The points in the baseline trace are the anchor points for the baseline curve, which was determined by averaging the raw signal for 2 s.

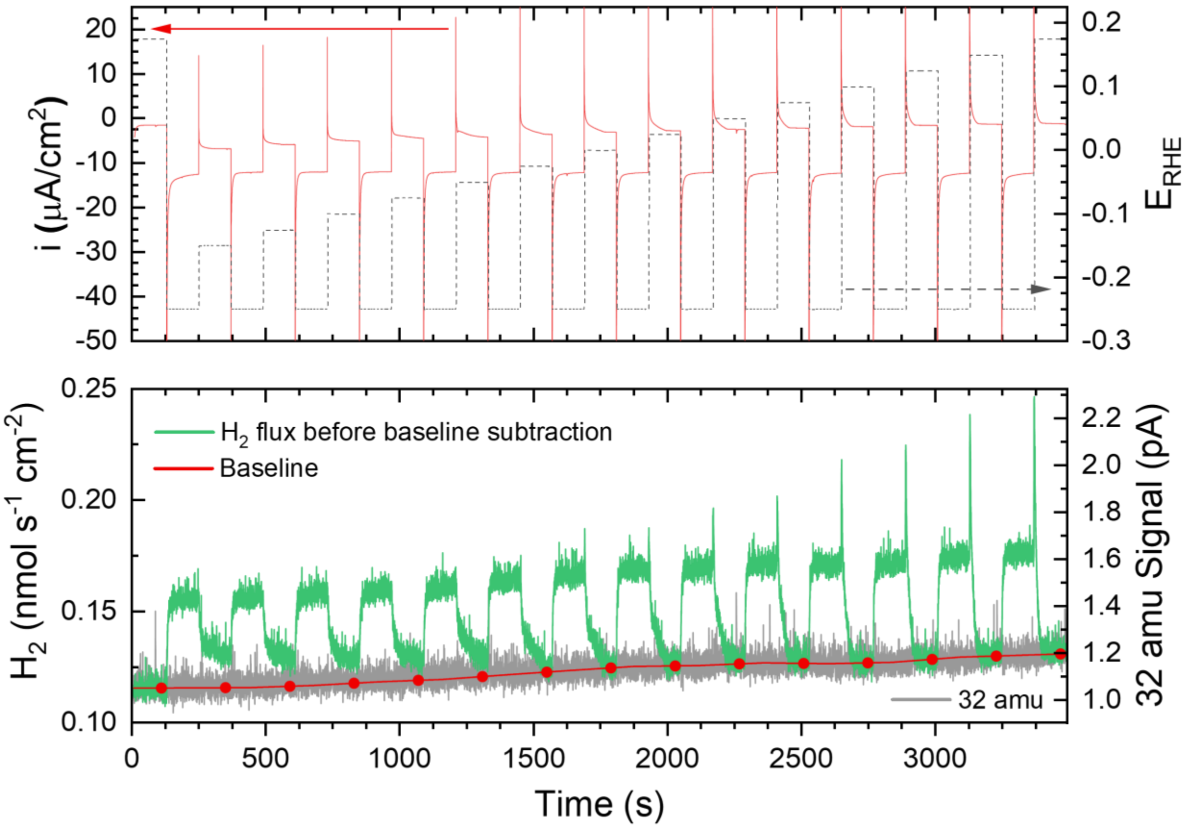

Figure S2. Positive step potential pulse measurements on Cu(111) in He saturated 0.1 mol L^{-1} $HClO_4$. The baseline was determined prior to conversion of 2 amu to H_2 flux, in order for the 32 amu baseline to serve as a guide (see the Experimental for more details).

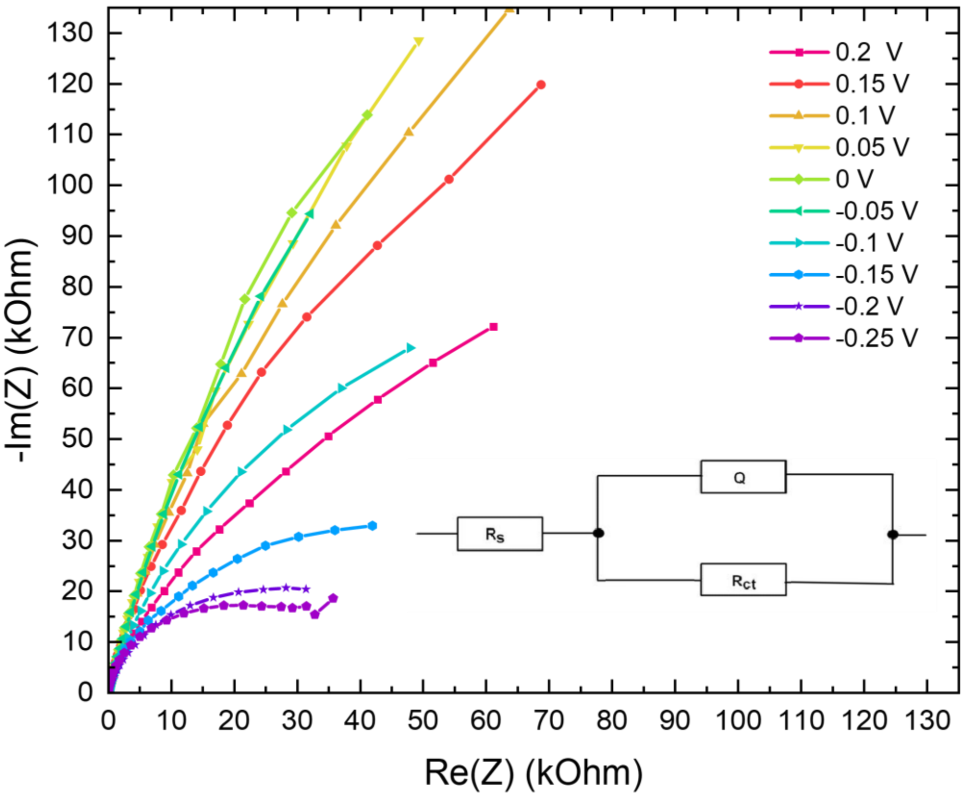

Figure S3. Electrochemical impedance spectroscopy on Cu(111) loaded in the EC-MS cell in He purged 0.1 mol/L $HClO_4$.

Table S1. Fitting constants for the simplified Randle circuit applied to the electrochemical impedance spectroscopy measurements shown in **Figure S3**.

E (V vs RHE)	R_s (Ohm)	Q (F $s^{\alpha-1}$)	α	R_{CT} (Ohm)
0.2	20.9 ± 0.6	9.3E-6 ± 3E-7	0.927 ± 0.006	120000 ± 7300
0.15	20.9 ± 0.6	8.1E-6 ± 2E-7	0.936 ± 0.005	258000 ± 21000
0.1	20.7 ± 0.5	8.5E-6 ± 2E-7	0.931 ± 0.004	345000 ± 35000
0.05	20.5 ± 0.5	9.4E-6 ± 2E-7	0.922 ± 0.004	426000 ± 53000
0	20.4 ± 0.4	1.1E-5 ± 2E-7	0.914 ± 0.004	472000 ± 67000
-0.05	20.2 ± 0.4	1.4E-5 ± 3E-7	0.905 ± 0.004	428000 ± 63000
-0.1	20.5 ± 0.4	1.5E-5 ± 3E-7	0.913 ± 0.004	153000 ± 10000
-0.15	20.2 ± 0.5	1.2E-5 ± 4E-7	0.944 ± 0.006	59000 ± 2700
-0.2	20.1 ± 0.3	2.1E-5 ± 4E-7	0.891 ± 0.003	47000 ± 1500
-0.25	21.2 ± 0.5	7.9E-6 ± 2E-7	0.972 ± 0.005	37000 ± 1000

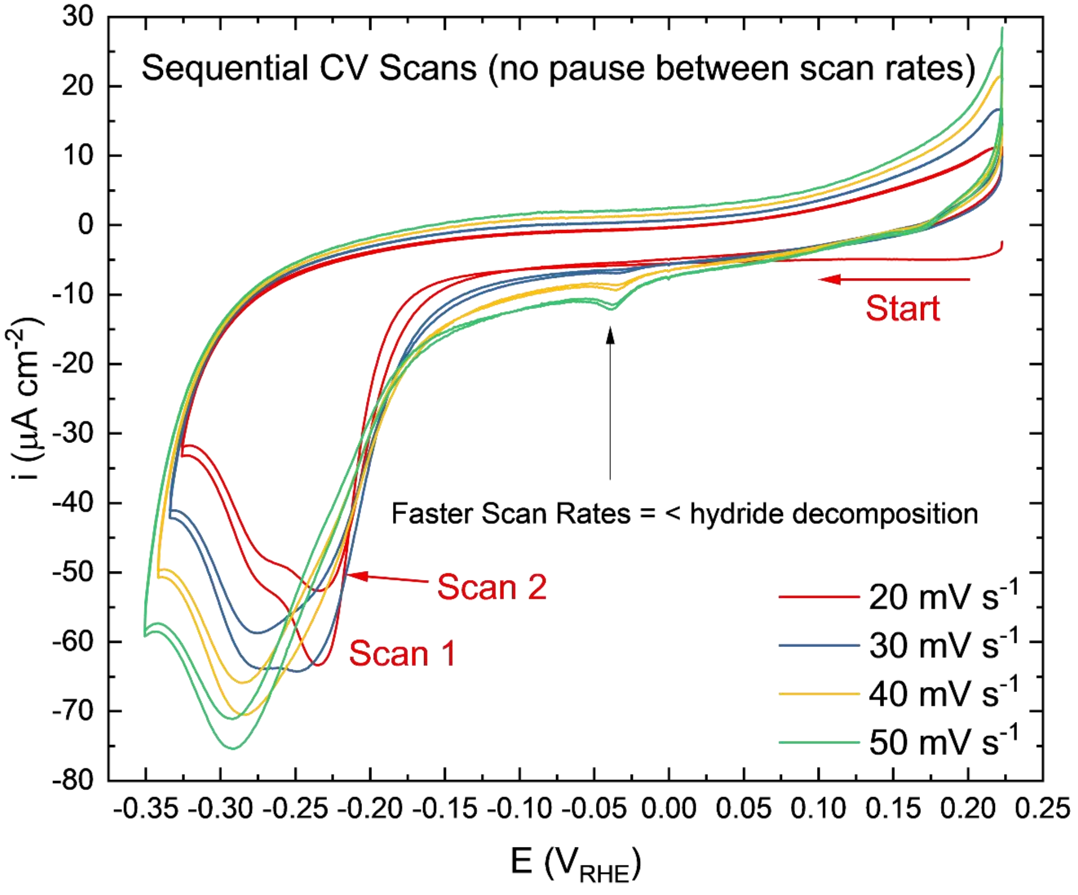

Figure S4. Cyclic voltammograms with no pausing between scan rates demonstrating incomplete decomposition of the hydride phase.

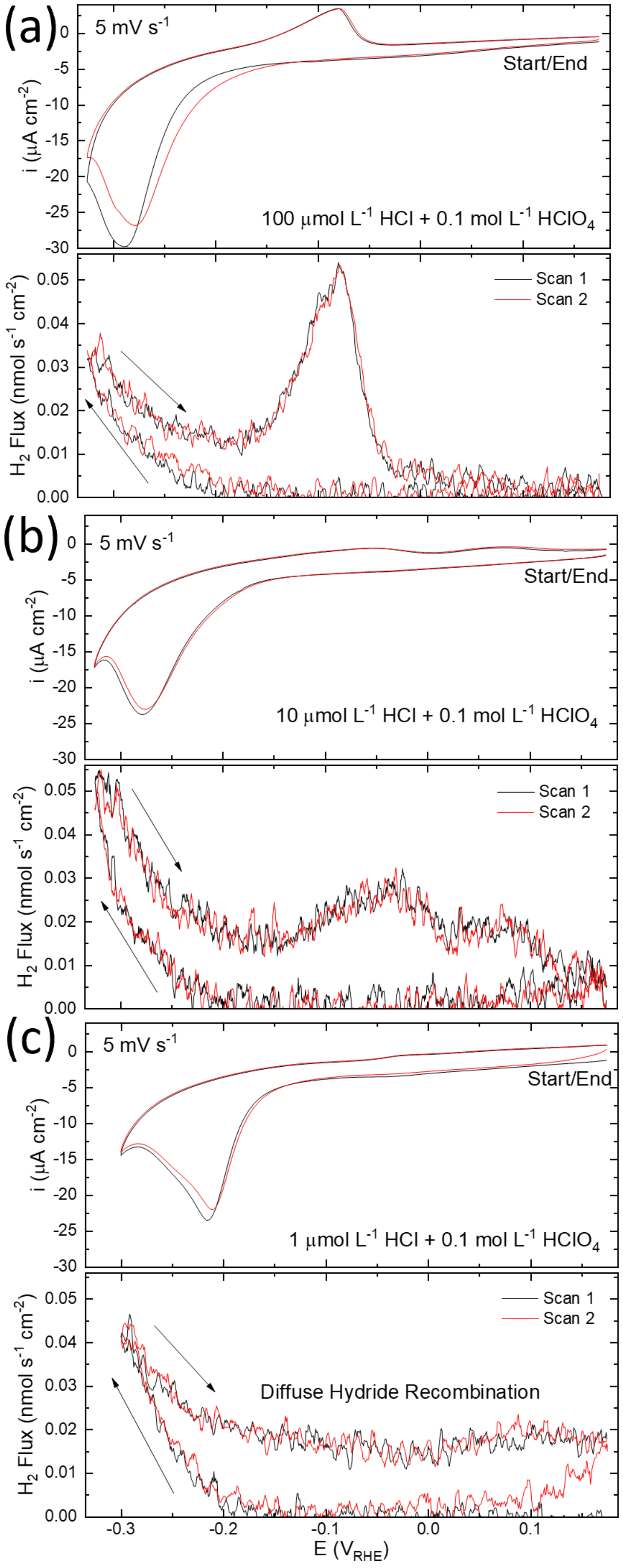

Figure S5. Cyclic voltammetry (2 complete sweeps) and corresponding electrochemical mass spectrometry of H_2 flux on Cu(111) in 0.1 mol L^{-1} $HClO_4$ containing (a) 100 µmol L^{-1} HCl, (b) 10 µmol L^{-1} HCl, and (c) 1 µmol L^{-1} HCl.

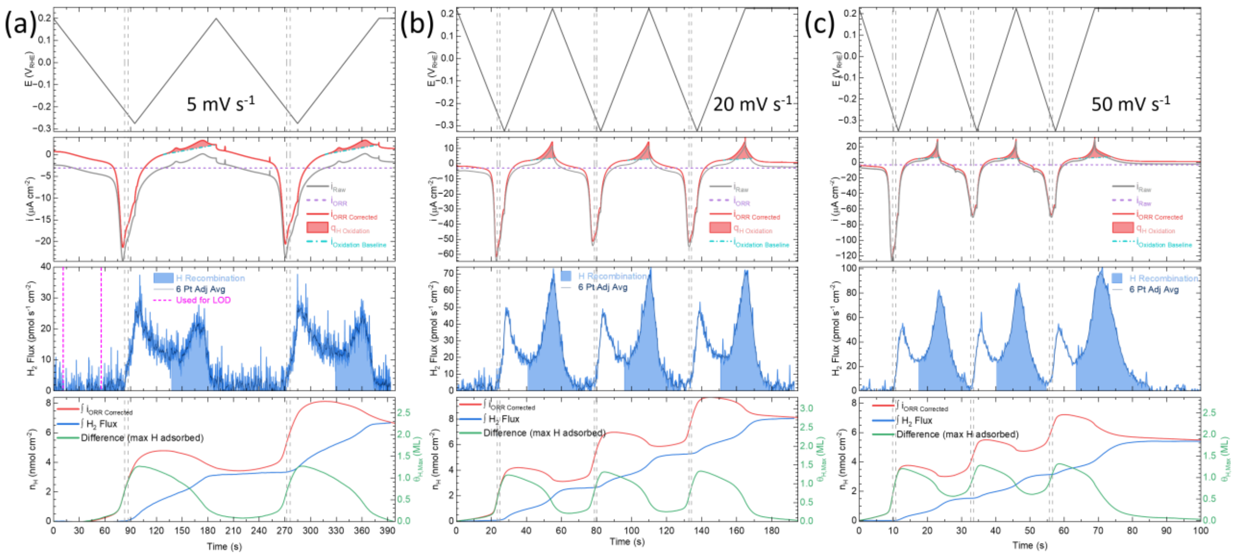

Figure S6. Traces of potential, current, and H_2 flux vs time from the (a) 5 mV s^{-1}, (b) 20 mV s^{-1}, and (c) 50 mV s^{-1} CVs shown in **Figure 1**. Integration of current and H_2 flux, as well as their difference, in the form of atomic hydrogen (nmol cm^{-2}) demonstrates the maximal possible coverage of H on the Cu surface as a function of potential. The magenta dashed vertical lines in (a) serve as an example of the baseline signal used to determine the limit of detection (LOD) of the electrochemical mass spectrometer. The two broad vertical dashed lines in (a-c) represent the region of HER onset for each sweep, **Table 2** indicates the potential of this onset.

Table S2. Comparison of the HER onset and max H surface coverage with scan rate from **Figure 2**.

**Scan Rate-Cycle (mV s^{-1}) (unitless)**	**LOD pmol s^{-1} cm^{-2}**	**E_{Onset} (mV)**	**H_2 flux (pmol s^{-1} cm^{-2})**	**Max H_{ads} (nmol cm^{-2})**	**θ_H (ML)**
5-1	5.5	-226 ± 6	6 ± 2	2.4 ± 0.2	0.82 ± 0.06
5-2	5.5	-220 ± 6	5.7 ± 0.9	2.3 ± 0.2	0.79 ± 0.06
10-1	4	-245 ± 6	5 ± 2	2.41 ± 0.08	0.82 ± 0.03
10-2	4	-231 ± 6	5 ± 1	2.11 ± 0.09	0.72 ± 0.03
20-1	6	-260 ± 10	6 ± 1	2.1 ± 0.06	0.71 ± 0.02
20-2	6	-260 ± 10	6 ± 1	2.63 ± 0.05	0.90 ± 0.02
20-3	6	-246 ± 10	6 ± 1	2.28 ± 0.06	0.78 ± 0.02
50-1	4	-290 ± 18	8 ± 5	2.20 ± 0.04	0.75 ± 0.02
50-2	4	-280 ± 18	7 ± 4	2.82 ± 0.02	0.96 ± 0.08
50-3	4	-280 ± 18	11 ± 4	2.93 ± 0.02	0.99 ± 0.08
Average					0.82 ± 0.02

* The hydrogen evolution reaction onset was defined as the limit of detection (LOD) of the ECMS. The LOD was found via the sum of baseline signal and 3 times the baseline's standard deviation via averaging the baseline signal over ≈ 30 s for each scan rate (**Figure S4**). The ± represents the standard deviation of the average.

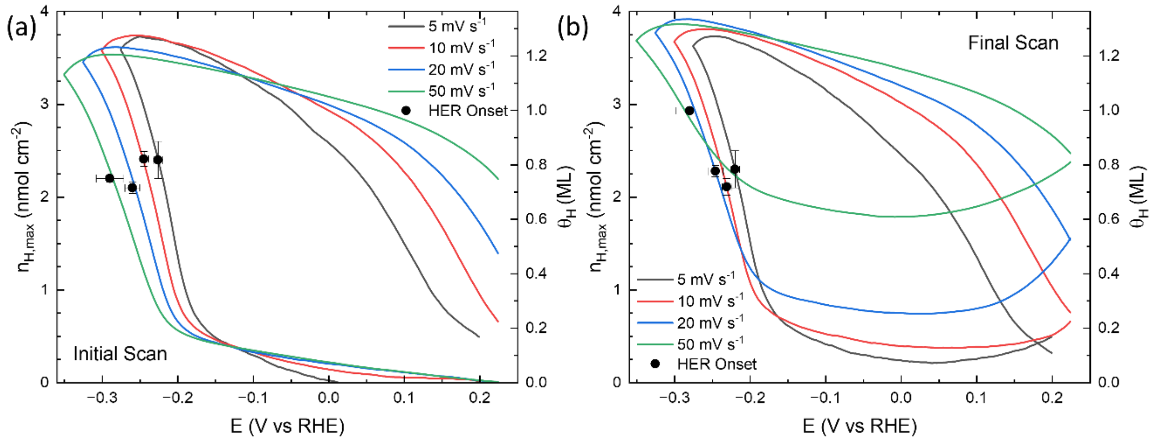

Figure S7. Maximal possible H coverage on Cu(111) as a function of potential for the (a) initial CV and (b) final CVs plotted in **Figure 2**. The onset information is tabulated in **Table S2**. The error bars represent the standard deviation from the average.

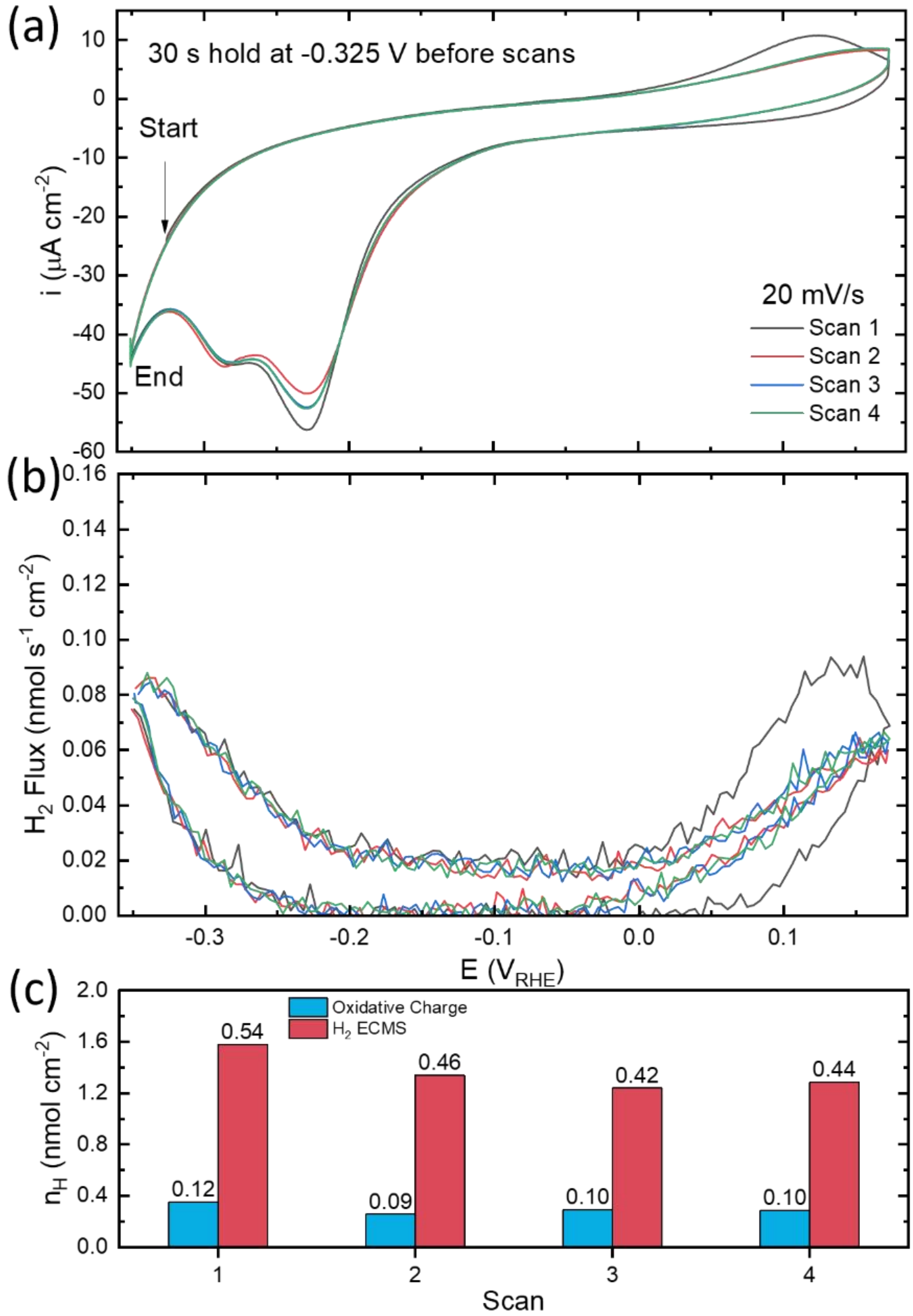

Figure S8. (a) Cyclic voltammetry of Cu(111) in He saturated 0.1 mol L^{-1} $HClO_4$ and the accompanying (b) H_2 flux measured by electrochemical mass spectrometry following an initial 30 s pretreatment at -0.325 V. The H_2 flux and oxidative peak on the anodic sweep were (c) integrated and converted to n_H (nmol cm^{-2}) and are labeled above the bar graph in terms of the H_{ads} fractional surface coverage θ_H.

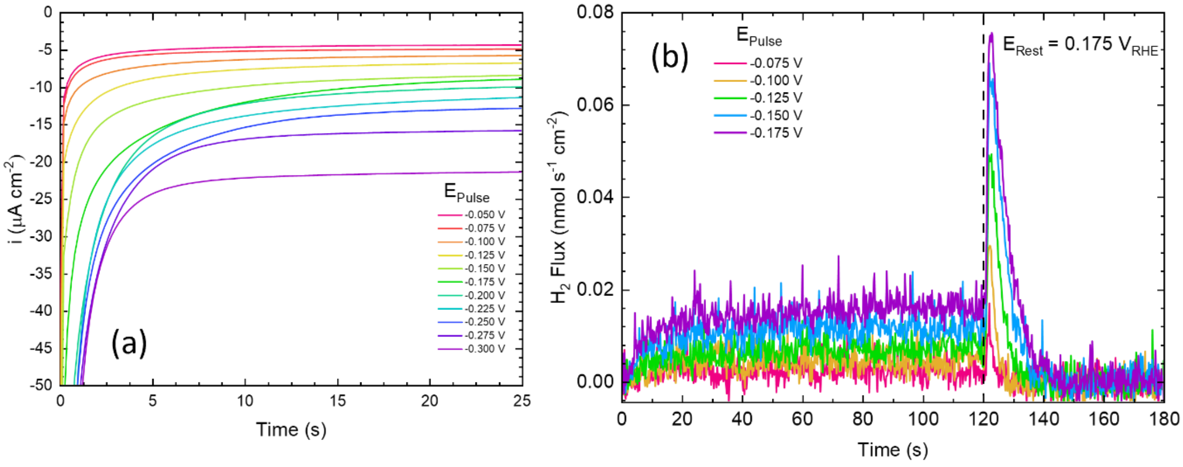

Figure S9. Zoom-in on (a) chronoamperometry during E_{Pulse} and (b) H_2 flux, during select E_{Pulse}, for negative step potential pulse measurements on Cu(111) in 0.1 mol L^{-1} $HClO_4$.

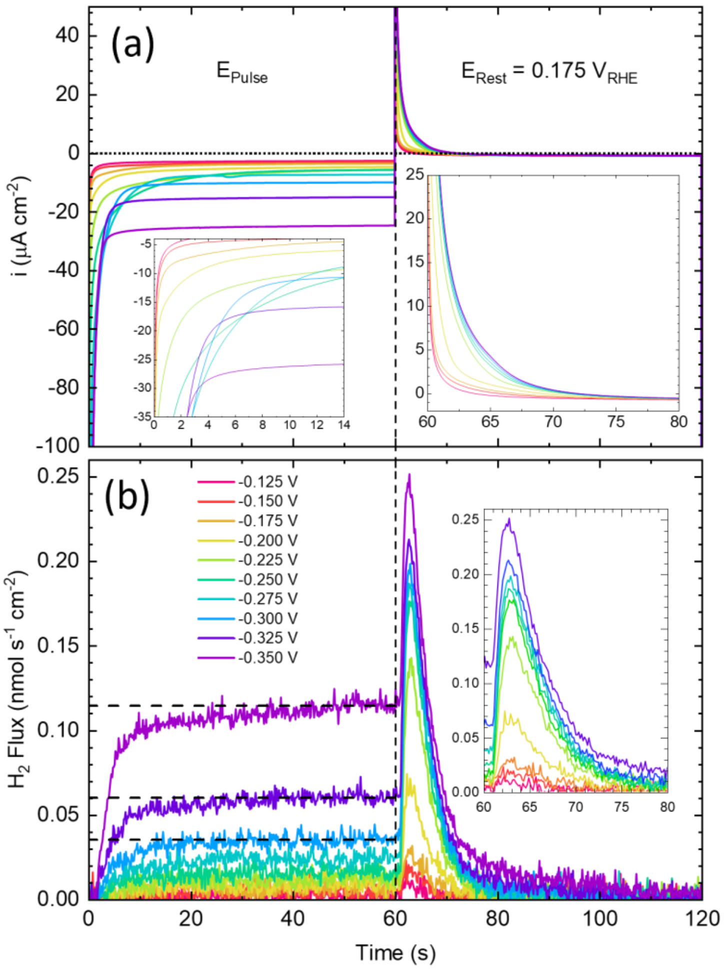

Figure S10. (a) Chronoamperometry and (b) H_2 flux during potential pulse measurements on Cu(111) in He saturated 100 µmol L^{-1} HCl + 0.1 mol L^{-1} $HClO_4$. The horizontal dotted line in (a) indicates zero current. The horizontal dashed lines in the H_2 flux plot indicate the steady state HER (HER_{SS}) flux at the three most negative E_{Pulse} as determined by averaging the last 30 s of the flux during E_{Pulse}. The vertical dashed line indicates the transition to E_{rest}. The inset magnifies the transient period at E_{Rest} associated with hydride decomposition to H_2 via H_{ads} recombination.

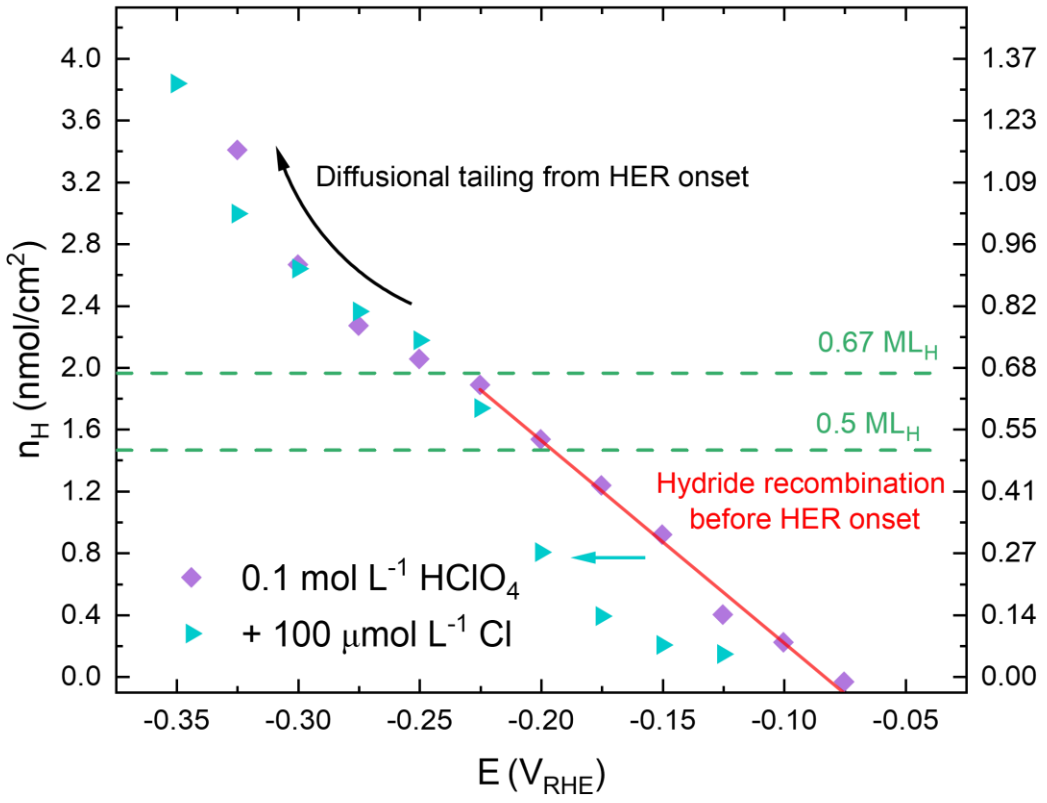

Figure S11. Integration of the H_2 flux at E_{Rest} from 120 s to 150 s for pulses in 0.1 mol L^1 $HClO_4$ (**Figure 4b**) or 60 s to 90 s for pulses in 100 µmol L^{-1} HCl + 0.1 mol L^{-1} $HClO_4$ (**Figure S10**) reveals an inflection near -0.225 V suggestive of a phase transition and onset of the HER.

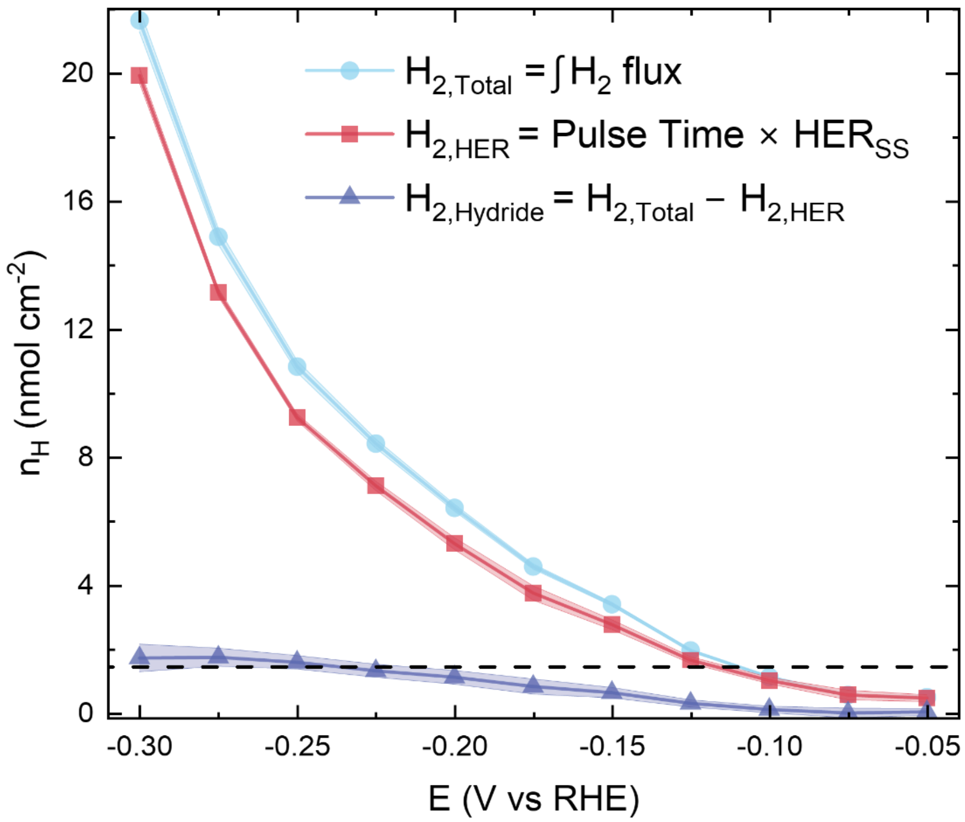

Figure S12. Quantities of H measured by the EC-MS during negative step potential pulse measurements in **Figure 4b**.

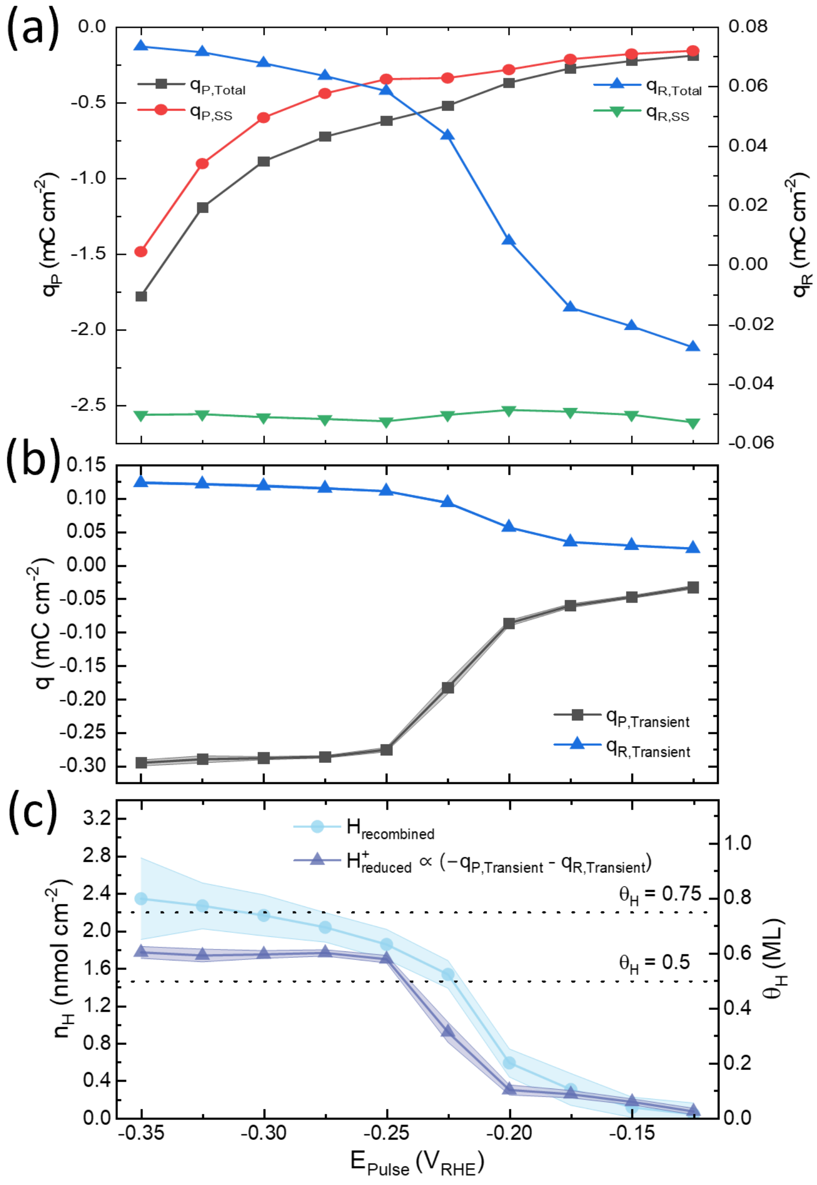

Figure S13. (a) The total, ($q_{P,Total}$ and $q_{R,Total}$) and steady state ($q_{P,SS}$ and $q_{R,SS}$) chronoamperometric charge and (b) their difference, the transient charge ($q_{P,Transient}$ and $q_{R,Transient}$), for E_{Pulse} and E_{Rest} during negative step potential pulse measurements in 100 µmol L^{-1} HCl + 0.1 mol L^{-1} $HClO_4$ (**Figure S10**). (c) Total H coverage determined from either the reduction wave, $H^{+}_{reduced}$, which was found by removal of $q_{R,Transient}$ (which is representative of Cl adsorption and capacitance) from $q_{P,Transient}$ or hydride recombination, $H_{recombined}$ from the EC-MS.

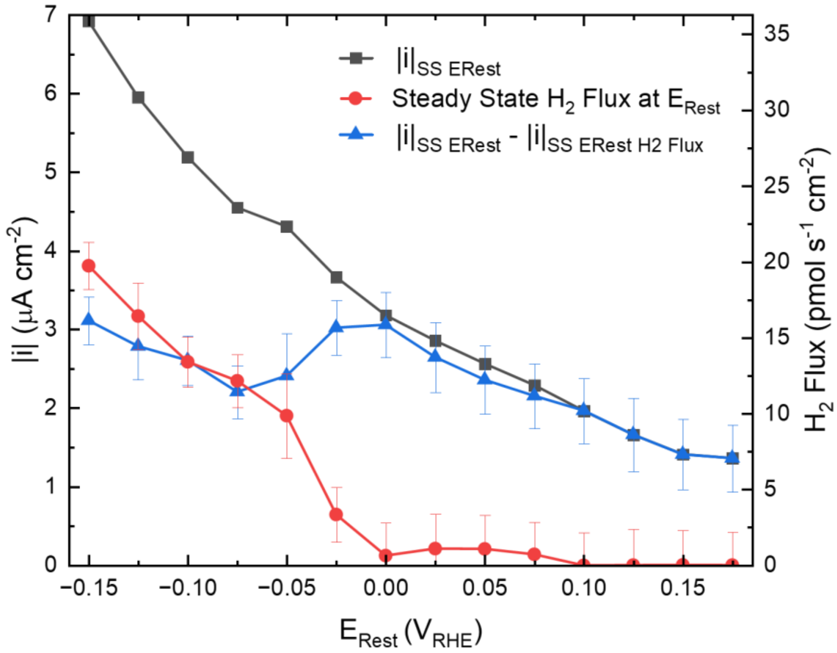

Figure S14. Steady state absolute current density and H_2 flux determined by averaging the last 5 s of E_{Rest} during positive step potential pulse measurements seen in **Figure 6**. The blue triangles represent the residual after converting the H_2 flux to current and subtracting it from the average steady state current. It nominally reflects the possible steady state trace oxygen reduction reaction and/or inaccuracies in the assumption that the last 5 s are representative of purely steady state processes.

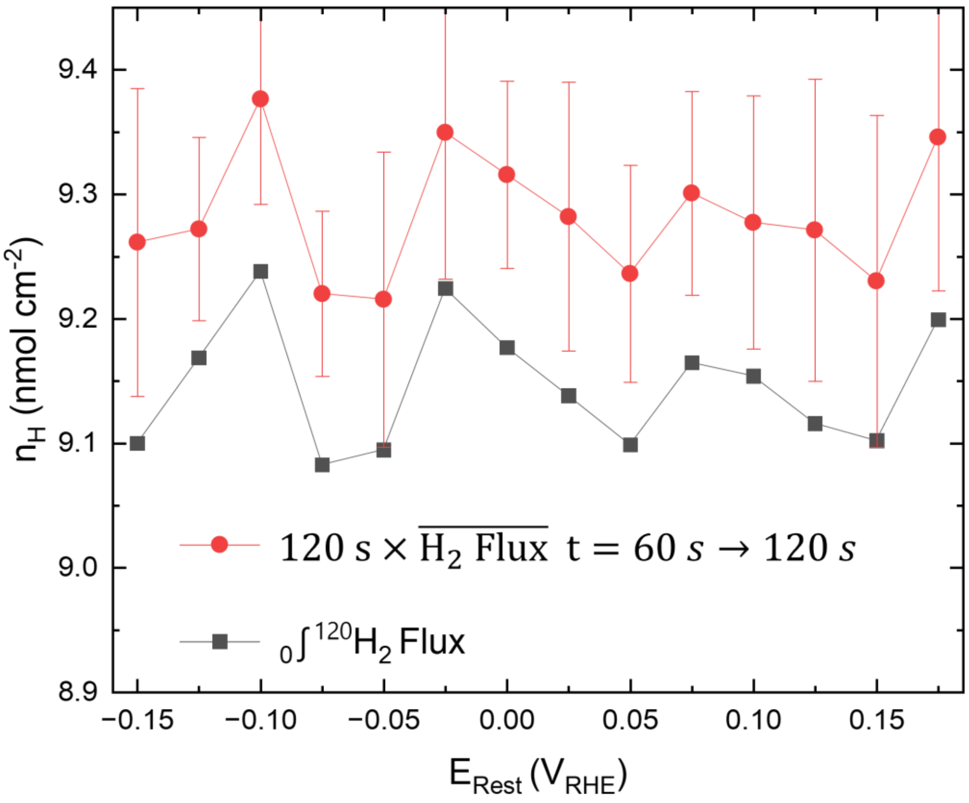

Figure S15. Total n_H and the contribution from steady state HER measured during E_{Pulse} for positive step potential pulse measurements shown in **Figure 6**. The difference between these two represents the "Residual H_2 from E_{Pulse}" presented in **Figure 7a**.

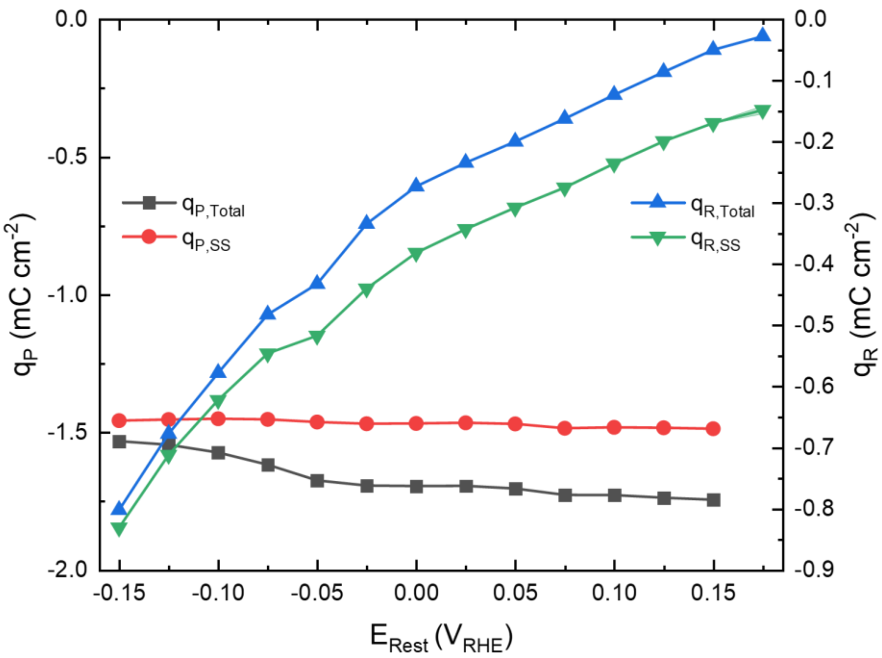

Figure S16. Total and steady state charges for E_{Pulse} (-0.25 V) and E_{Rest} during positive step potential pulse measurements.
